# Causal effects of social media use on self-esteem, mindfulness, sleep and emotional well-being: a social media restriction study

**DOI:** 10.3389/fpubh.2025.1548504

**Published:** 2025-05-30

**Authors:** Matthias Maerevoet, Marlies Van de Casteele, Eowyn Van de Putte, Dries Debeer, Kristof Hoorelbeke, Maarten Vansteenkiste, Ernst H. W. Koster

**Affiliations:** ^1^Department of Experimental Clinical and Health Psychology, Ghent University, Ghent, Belgium; ^2^Department of Developmental, Personality and Social Psychology, Ghent University, Ghent, Belgium; ^3^Faculty of Psychology and Educational Sciences, Research Support Office, Ghent University, Ghent, Belgium

**Keywords:** social media use, social media reduction, self-esteem, sleep, emotional well-being

## Abstract

**Introduction:**

The question whether social media use (SMU) has a causal influence on mental health sparks a lot of interest. Empirical research to date shows no consensus on the causal effects of SMU on mental well-being. Therefore, the present study assessed if experimentally implemented restrictions in SMU led to improvements in well-being outcomes using a combination of self-report and passive sensing data.

**Methods:**

After a 2 week baseline phase, participants (*M* age = 21.42 years) were randomly assigned to an experimental (*N* = 35) or a passive control (*N* = 32) condition. Participants in the experimental condition were asked to limit their SMU to a maximum of 30 min (divided across their preferred apps) per day for two consecutive weeks, while participants in the control condition were instructed to continue their SMU as usual. After the intervention phase, participants in both conditions were followed up for 2 weeks during which all restrictions were removed. During the experiment, we monitored self-esteem, mindfulness, sleep, and emotional well-being.

**Results:**

Results indicate a main effect of time for most outcomes, but the implemented SMU restriction did not moderate these effects.

**Discussion:**

In conclusion, this study found no benefits from a temporary social media reduction on mental health outcomes.

## Introduction

Over the last decade, social media use (SMU) has taken a central role in the way we interact and communicate with each other. Especially in youth, SMU plays a major role in social communication with image-based apps such as Instagram and TikTok being particularly popular (1). Given the centrality of online engagement in daily life, this prompted an inquiry into the potential impact of SMU on the mental health of youth (2). Although most of the research has focused on adolescents, growing evidence suggests that SMU also impacts adults’ mental health, particularly among young adults (3). Today, the general consensus of cross-sectional studies is that more time spent on social media is weakly associated with reduced levels of well-being or ill-being (4–7). However, some research indicates a positive relationship between SMU and (social) well-being (8, 9), while others find no significant associations between SMU and mental health at all (8). Valkenburg et al. (2) consider this a “bag of mixed findings” and highlight the need for more sophisticated methodologies, such as longitudinal and experimental studies, to more effectively investigate the (causal) relationship between the two (10, 11). Despite progress in the research field over the last decade, results on causal effects of SMU remain inconclusive (7) and studies testing whether reducing SMU improves mental health are particularly scarce (12).

To contribute to this expanding body of research and investigate causal effects of SMU on mental health, the present study combines an experimental design with a longitudinal follow up. In particular, a convenience sample of bachelor and master students were instructed to reduce their SMU to 30 min per day while subsequently tracking changes in mental health. This approach addresses key gaps identified in previous social media reduction studies. One limitation is that most studies focus on a narrow range of mental health outcomes, leading to a fragmented understanding of the overall effects of social media reduction (2, 13). To overcome this, we adopt a broad definition of mental health, considering not only the absence of psychopathology or ill-being (e.g., depression, anxiety, stress), but also incorporating positive aspects of well-being (2, 14), such as the presence of positive emotions, mindfulness, and self-esteem (14). Given the essential role of sleep in mental health (15) and its association with SMU (16), we were also interested in exploring how limiting SMU might influence sleep, thereby situating it within the broader umbrella of mental health. Herein, passive sensing data is crucial for this assessment, as it allows continuous, non-invasive monitoring without disrupting sleep or adding participant burden through daily questionnaires.

Second, although prior experimental studies asked participants to limit or stop SMU during the experimental phase, most did not include a reliable measure of usage duration, such as a device-based tool or app, making it difficult to accurately assess compliance with restrictions (17, 18). To address this, the present study employed an app installed on the smartphones of participants (i.e., MobileDNA) (19) to objectively track their SMU across all study phases, thereby allowing to reliably check compliance. Lastly, few experimental studies included multiple follow-up measurements, leaving it unclear whether the observed effects persist only during the intervention phase, immediately afterward, or over a longer term (18, 20). For example, while an imposed restriction may lead to a temporary reduction in SMU, young people might bounce back by overusing at a later moment (21). Conversely, an intervention might not show immediate effects due to initial “withdrawal-like symptoms” in some participants, with positive outcomes only emerging over a longer period (22). To overcome this gap, hypothesized study outcomes were measured throughout the study and during a two-week follow-up.

### Longitudinal studies and temporal sequence

Longitudinal studies, compared to cross-sectional studies, offer more insight into causal pathways by tracking changes over time, allowing researchers to explore correlations and infer the potential direction of the relationship between SMU and mental health (10, 11). Despite an increase in longitudinal research, empirical evidence remains inconclusive. A systematic review by Tang et al. (23) of 35 longitudinal studies (2005–2020) on screen time and youth psychopathology found small to very small effect sizes for depressive symptoms. No significant longitudinal associations were found for other internalizing symptoms such as anxiety, or general internalizing problems. Among the eight studies specifically examining SMU, evidence was mixed, with some studies suggesting a small association between SMU and later depressive complaints, internalizing problems, and psychological distress, but not with symptoms of anxiety. Similarly, a narrative review (24) of 14 longitudinal studies (2006–2019) on youth’s SMU specifically found limited evidence that the frequency of SMU is significantly associated with more ill-being. However, the longitudinal studies involving college students showed that passive SMU (e.g., browsing) was more consistently linked to increased depressed mood compared to active use (e.g., liking, posting, commenting) (25). Moreover, the differences in time lags across studies might help to explain the mixed findings across longitudinal studies. As argued by Haidt et al. (26) in their literature review, it is not yet clear what time interval is most appropriate for examining changes between SMU and mental health. Among the 26 longitudinal studies reviewed, 13 reported a significant effect, while the other 13 did not. Studies that used an interval of 1 month or longer appeared to be more likely to find a significant effect.

The longitudinal relationship between SMU and well-being indicators remains less well-established compared to its link with psychopathology and ill-being. A meta-analysis of both cross-sectional and longitudinal studies on the relationship between SMU and positive aspects of well-being (e.g., social well-being, happiness, self-actualization) found no evidence of longitudinal effects of SMU on well-being. Additionally, no longitudinal studies examining the relationship between SMU and mindfulness and self-esteem were available for inclusion in the meta-analysis (27). For mindfulness specifically, most studies focused on low trait mindfulness at time one predicting problematic SMU at time two (28). One three-wave longitudinal study partially supported the hypothesis that greater SMU predicted lower mindfulness, which in turn predicted more problematic SMU (29). Longitudinal studies investigating the relationship between social media use and self-esteem have also produced mixed findings regarding the direction of the association. When effects are observed, they are generally small in magnitude (30). Lastly, regarding sleep outcomes, a recent meta-analysis found a modest, but significant association (*r* = −0.12, *p* < 0.05) between SMU and later sleep health across five studies, while two studies examining the reverse relationship found a non-significant effect (*r* = −0.05, *p* = 0.06). The authors concluded that SMU’s impact on sleep was weaker compared to traditional media (31).

While the prevailing view suggests that SMU leads to reduced well-being and psychological complaints, some research argues for a reverse causal relationship, proposing that individuals with pre-existing psychological conditions may be more likely to engage in frequent or problematic SMU (32). For instance, as reviewed by Hartanto et al. (20), several longitudinal studies provided evidence that depression precedes increased SMU, while SMU does not appear to predict depressive symptoms. The authors further suggested that a bidirectional relationship may exist, in which depression leads to compensatory SMU, which in turn exacerbates depressive symptoms (33). Other studies (23) found little evidence for a reverse temporal sequence where psychopathology predicts SMU. Importantly, while longitudinal designs can help to establish temporal ordering, they cannot exclude potential confounding variables. Therefore, experimental research is needed to more accurately determine direction of effects and causality.

### Experimental evidence and causality

To establish causality, experimental designs are considered the gold standard. In the context of SMU, experimental approaches can take various forms. However, since experimentally increasing SMU is not an ethical option due to potential negative effects, most studies compare a condition where SMU is reduced or stopped completely with a control condition where SMU is not manipulated. While some studies have examined reductions in overall screen time on well-being (34, 35), this study focusses on interventions that specifically target SMU, leaving other screen-based activities (e.g., messaging, watching television) unaffected. We chose to focus on SMU due to its interactive, algorithm-driven, and highly personalized nature. These features make social media use especially engaging and potentially habit-forming. They also explain the prominent role in ongoing societal and academic debates and highlight the need for social media-specific evidence.

Radtke et al. (18) reviewed the effects of digital detoxes, defined as a period during which the use of digital devices (e.g., tablets, smartphones) or (categories of) apps (e.g., social media, TikTok, Instagram) is restricted. Out of all 21 digital detox studies, 12 addressed the effects of reducing SMU specifically on indicators of mental health, with some focusing on reducing the use of specific apps (e.g., Facebook or Instagram; *k* = 7), the most prominent apps (*k* = 2) or social media in general (*k* = 4). All interventions reported successful reductions in SMU during the intervention phase, but only three studies have used an application to objectively track compliance (36–38). Most studies looked at indicators of mental health (i.e., depression, anxiety, stress) or hedonic well-being (i.e., positive, and negative affect), with some including indicators of eudaimonic well-being, mostly self-esteem or life satisfaction. Six out of 12 did not find an effect on the examined indicators of mental health. Of the studies that did find an effect, most were positive, but in some studies only for a certain subgroup (39) or on only one of a subset of the examined outcomes (38). Few report negative effects of reductions in SMU, such as on affective well-being (37) or on connection (21). This review also included two studies examining the effects of a screen time detox on sleep, one using self-reports and one using passive sensing data, neither of which found significant effects; however, these studies did not specifically address SMU.

Similarly, Plackett et al. (40) conducted a systematic review with a total of 23 studies on the impact of SMU interventions on mental health, nine of which overlap with those reviewed by Radtke et al. (18). Different from Radtke and colleagues, they focused specifically on (young) adults (i.e., 70% of studies used university students), SMU specifically, and adopted a broader definition of SMU interventions including both reduction studies, full abstinence, and therapy-based interventions. The authors concluded that 39% of the studies found improvements in mental health, 30% found mixed evidence, and 30% found no effects at all. Of the studies that found an effect, most of them showed medium to large effect sizes. Improvements in depressive symptoms were most prominent, with 70% of the studies that assessed depressive symptoms showing beneficial effects of reduced SMU. For other outcomes, mixed evidence was found. Regarding loneliness, some studies found that an SMU intervention decreased the feeling of loneliness (41) while others found an increase (42). Mindfulness on its turn was found to increase in one study (43) and decrease in another study (44). Their review concluded that some SMU interventions are effective in improving mental health, particularly for depression and when interventions based on cognitive behavioral therapy were used. Although they found that reduction studies showed fewer systematic improvements in mental health, they argued that maintaining adherence might be challenging for participants and was often not clearly tracked in these studies.

Most recently, Ferguson (45) published a meta-analysis of 27 studies conducted between 2013 and 2023, demonstrating that, across experiments, experimental interventions did not have a significant effect on well-being (*d* = 0.088, *p* = 0.104).

Although strong causal claims about the relationship between SMU and mental health are often made (46), recent reviews and meta-analyses reveal substantial variability in outcomes, underscoring the need for further experimental and longitudinal studies. Future experimental studies should aim to improve adherence tracking, integrate more objective measures, and carefully consider the processes targeted by interventions to better isolate the causal pathways linking SMU to mental health outcomes. These considerations form the premise for the current study’s design and objectives.

### The present study

The present study applied an experimental design to examine the causal influence of SMU on a variety of mental well-being outcomes (self-esteem, mindfulness, sleep, internalizing complaints, positive and negative affect). We recruited participants who reported high SMU (daily SMU of minimum 2 h per day) since previous research showed that changes in well-being particularly arise in high frequent social media users and less in low social media users (approximately 30 min to 1 h per day) (47–49). Participants were randomly assigned to a control or experimental condition; they were monitored for 6 weeks and participants in the experimental condition were asked to reduce their SMU during the intervention. As social media plays a central role in society, we chose to apply a SMU *reduction* instead of complete SMU *abstinence* to make the intervention more pragmatic and feasible to incorporate in daily life. Moreover, the goal of this study was mainly to reduce passive SMU (e.g., consuming content without direct interaction) and not active SMU (e.g., engaging with others and creating content), because active use is shown to enhance well-being through social support (50).

Despite the mixed findings reported in this literature, a recent meta-analysis indicates that the duration of the social media manipulation should last 2 weeks or more to observe beneficial effects (51). As our study design manipulated social media use for 2 weeks, we expected to observe similar positive findings of reducing SMU. Rather than one overall mental well-being indicator, we selected a set of highly relevant outcomes related to it. While they cannot be seen as independent from each other and while they do not constitute a complete list to fully encompass the concept of mental well-being, we believe that possible changes related to SMU in these outcomes can have direct implications on mental well-being.

## Materials and methods

### Participants and procedure

**Figure 1:**
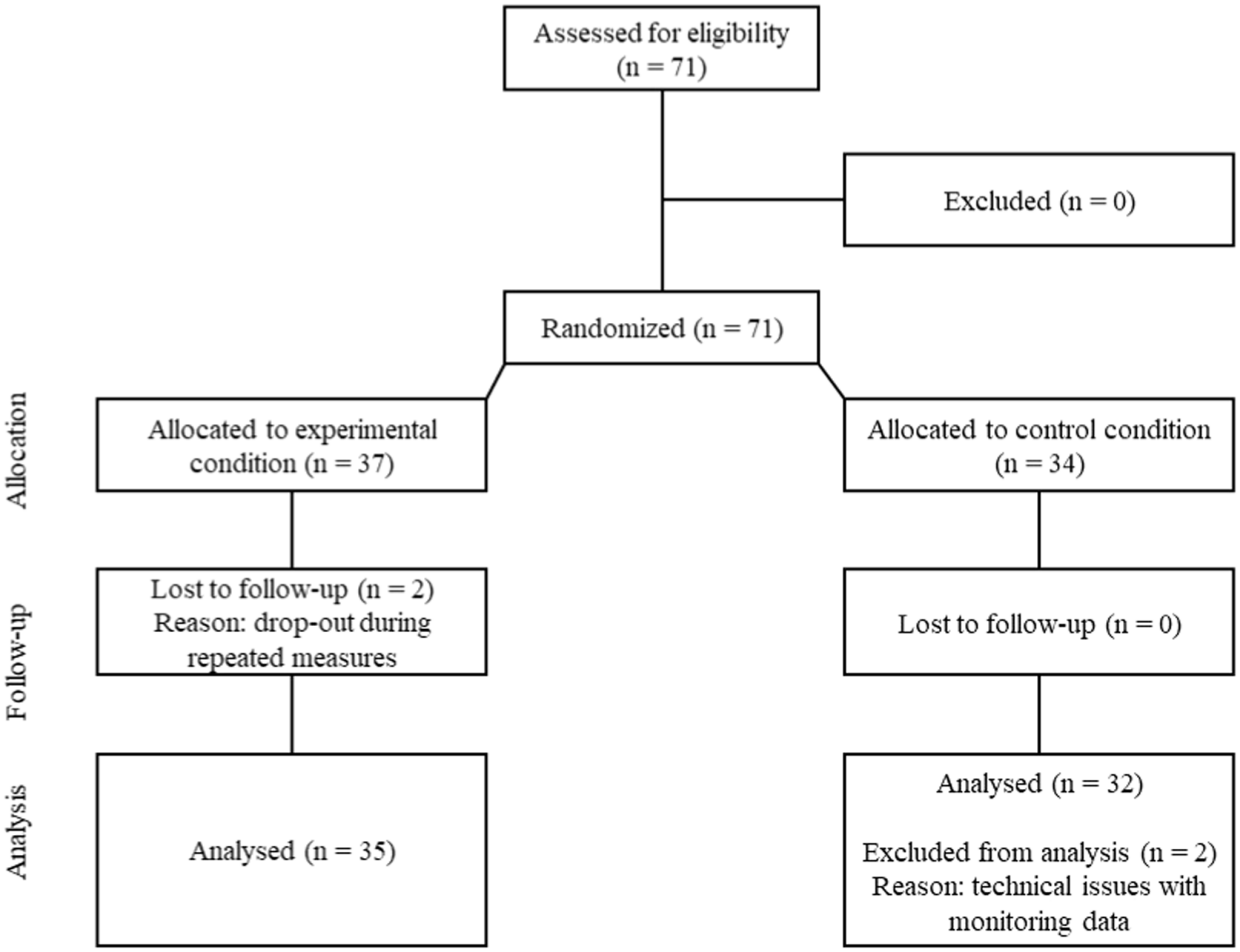
Consort diagram.

University students between the ages of 18 and 26 years were recruited through flyers, social media and MoodSpace, an online mental health platform that informs students about mental health. Inclusion criteria for the study were (1) being a student at a Flemish higher education institution; (2) having an Android smartphone; (3) daily SMU of minimum 2 h per day. In order to reduce possible confounding variables on our mental health outcomes and objectively monitored sleep variables, exclusion criteria were using sleep medication, current symptoms of depression, pregnancy, having children, and shiftwork. In total, 71 students enrolled in the study with a drop-out of 4 participants, resulting in a final sample size of 67 participants (see Figure 1). All participants signed a written consent form at the beginning of the study and received a monetary reimbursement of €40 after completion of the follow-up questionnaires. The study was approved by the Ethical Committee of Ghent University.

**Figure 2:**
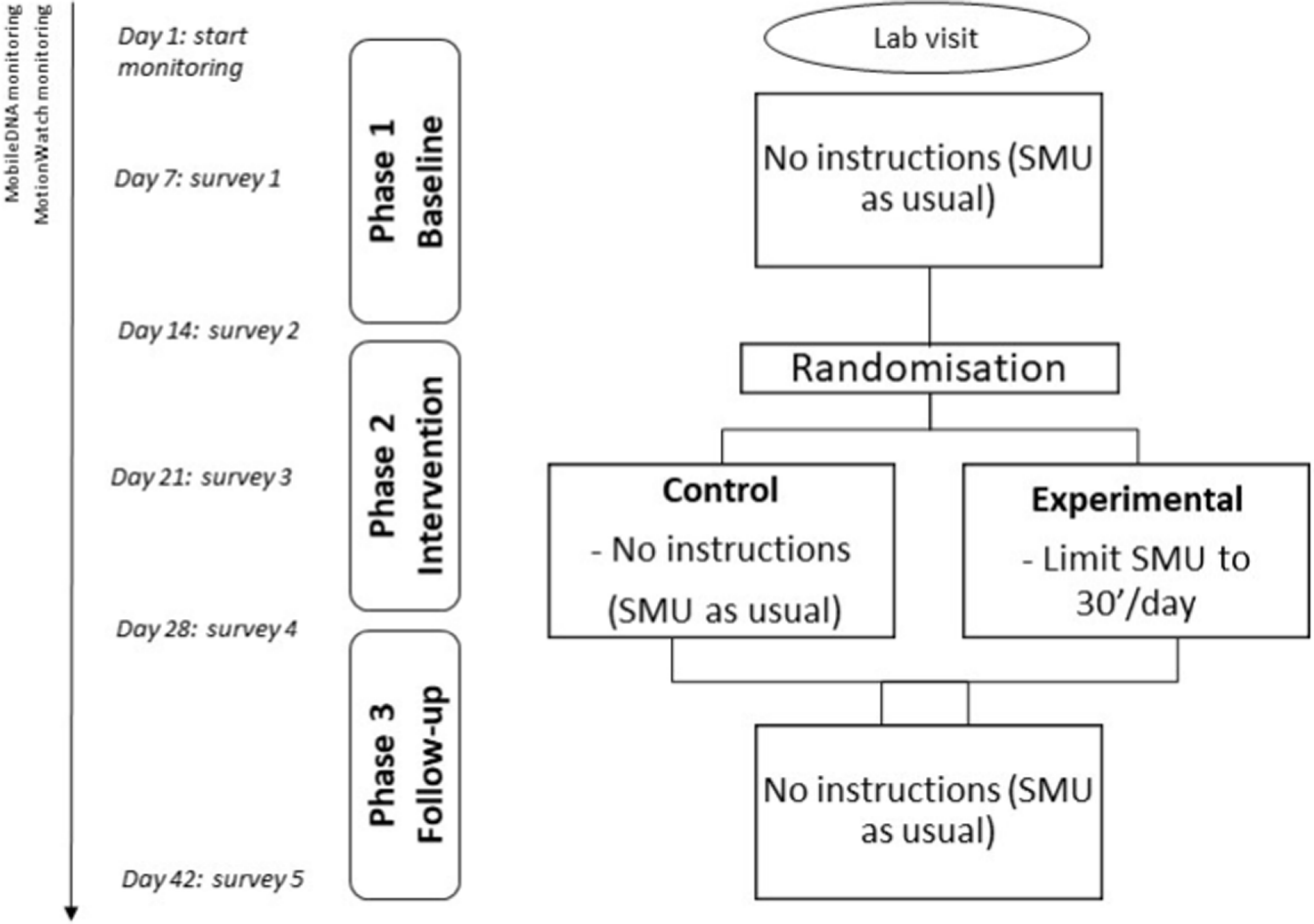
Graphical representation of study design.

The study was spread over a 6-week period in May–June 2022 and was divided into three phases of 2 weeks (see Figure 2). First, the MobileDNA app (i.e., smartphone tracking) and the MotionWatch (i.e., sleep tracking) were installed during a lab visit. Throughout the entire course of the study, participants’ sleep, and smartphone use were tracked. As depicted in Figure 2, students filled out questionnaires regarding their self-esteem, mindfulness, sleep and emotional well-being at five measurement occasions.

The first phase included a 2-week baseline period, in order to gain insight into pre-intervention SMU and baseline measures of the participants. During this phase, no explicit instructions were given regarding SMU. Participants were informed that the aim was to measure their SMU and sleep as naturalistic as possible, so no intentional behavioral changes were required. No further details were provided about the hypotheses and the upcoming restriction phase to limit the influence of *a priori* hypotheses and self-selection biases in recruitment.

At the start of phase 2, participants were randomly assigned to either the experimental condition (i.e., SMU restriction; *n* = 35) or the control condition (i.e., SMU as usual, *n* = 32), using a double-blind procedure. In the experimental condition, participants were instructed to reduce their SMU to 30 min per day. Smartphone settings (e.g., digital well-being and digital balance) were used to temporarily place timing restrictions on common social media apps (e.g., Instagram, Facebook, TikTok…). To enhance participants’ autonomy, participants could choose how they wanted to divide the 30′ time spent on the different platforms, as long as the total time was limited to 30′ distributed over the different media. For example, participants could choose to divide the 30′ over 3 different apps that they each could use for 10′ or they could decide to only use 1 app for 30′. All other apps were restricted to 0′. When participants reached the maximum time limit, the app shut down and a notification appeared that they reached the time limit of the day. No restrictions were placed on communication apps (e.g., WhatsApp, Messenger) as we did not want to reduce social support. To ensure that people adhered to the intervention, SMU was monitored with the mobileDNA app during the study period. In the control condition participants were instructed to keep on using their smartphone as they usually would.

At the beginning of phase 3, the intervention ended, and all restrictions were removed. Participants in the experimental group were thus informed that they were allowed to freely use all social media apps. During this 2-week follow-up phase, SMU and sleep patterns were still monitored to investigate sustained influences of the intervention. At the end of the follow-up phase, the sleep and social media monitoring devices were removed.

### Measures

#### MobileDNA

Time spent on social media with their smartphone was objectively monitored through the logging application MobileDNA (19). MobileDNA, an Android-exclusive smartphone logging app developed by Ghent University, is publicly available. Once installed, it records various aspects of smartphone usage, including the apps utilized, time spent on each app, frequency of phone checks, and notifications received from each app. On one hand, MobileDNA serves as a tool for individuals to monitor (i.e., get feedback on) their personal smartphone habits, which are accessible via the app’s dashboard feature. Conversely, it can also function as a research instrument, providing raw data for analysis. In this study, the dashboard functionality was disabled to assess participants’ smartphone usage in a naturalistic context. By disabling this functionality, participants did not receive feedback on their smartphone usage. As such, the app had minimal interference with their usual smartphone behavior. In this study, the MobileDNA data was mainly used to check adherence to the social media restrictions used in phase 2, as well as continuation of effects of the experimental manipulation on SMU during phase 3.

#### Global explicit self-esteem

The Rosenberg self-esteem scale (RSES) (52) (Dutch translation) (53) is a well-established self-report questionnaire consisting of 10 statements that assess global feelings of self-worth or self-acceptance (*α* = 0.88). Participants need to rate on a 4-point Likert scale whether they strongly agree, agree, disagree, or strongly disagree with each of the 10 statements during the past 2 weeks (e.g., “I feel that I am a person of worth, at least on an equal plane with others”). Higher average total scores reflect higher self-esteem.

#### Mindfulness

The Mindfulness Attention and Awareness Scale (MAAS) (54) is a 15-item self-report questionnaire that measures mindfulness, defined as the ability to be fully present and allocate the attention and awareness to what occurs in the present moment (e.g., “I rush through activities without being really attentive to them”). Participants are asked to indicate how frequently they experienced each of the 15 statements during the past 2 weeks using a 6-point Likert scale from 1 (almost always) to 6 (almost never). The MAAS has shown good internal consistency (*α* = 0.87) and good psychometric properties (55). Higher average total scores reflect higher mindfulness.

#### Sleep

The MotionWatch 8 (MW8; CamNtech, Cambridge, United Kingdom) is an actigraphy watch (i.e., accelerometer) that measures sleep parameters in a reliable and ecologically valid way (56–58). Although polysomnography is the most robust and valid measurement of sleep, actigraphy is currently accepted as a valid, practical (i.e., mobile) alternative to polysomnography, allowing for long-term continuous sleep assessments in a naturalistic setting (56, 58, 59). The baseline, intervention, and follow-up phase all consisted of 2 weeks as the MW8 needs to be worn for 2 weeks to obtain a valid measurement of the sleep pattern. The sleep epochs that are recorded every 60 s were transferred from the watch to a computer using the Motionware software (CamNtech, Cambridge, United Kingdom) for further analysis. The MW8 monitoring resulted in an estimation of different sleep quality parameters including sleep latency, sleep duration, actual time awake, efficiency and fragmentation (indicator of sleep quality) (60) that were automatically calculated with the Motionware software (57, 61). Sleep latency is the period required for sleep onset after retiring to bed. Sleep duration is the difference in hours and minutes between sleep onset and sleep offset. Actual time awake is the total time spent in wake according to the epoch-by-epoch wake/sleep categorization. Sleep efficiency is the percentage of time in bed spent sleeping. Finally, fragmentation is the sum of the “mobile time (%)” and the “immobile bouts smaller of equal to 1 min (%).” This is an indication of the degree of fragmentation of the sleep period and can be used as an indication of sleep quality.

Next, to the objective data on sleep, we used the Pittsburgh Sleep Quality Index (PSQI) (62) to obtain measures on self-reported sleep quality. However, due to a technical error, we were unable to analyze the results of the PSQI properly. Hence, we decided to exclude the data from this measure in our study.

#### Internalizing complaints

The Depression Anxiety Stress Scale (DASS) (63) is a self-report questionnaire that captures internalizing complaints (i.e., depressive, anxious and stress-related). The complete questionnaire has 42 items that can all be answered on a four-point Likert scale ranging from 0 (not at all or never applicable) to 3 (certainly or usually applicable). For this study we used the short form that is translated to Dutch and has 21 items (DASS-21) (64). Equally divided over seven questions, each of the three internalizing complaints are represented in this questionnaire and are retrospectively evaluated over the past week. The three subscales showed good internal consistency (depression subscale: *α* = 0.83; anxiety subscale: *α* = 0.83; stress subscale: *α* = 0.85). Previous research has indicated good to excellent psychometric properties in both clinical and non-clinical populations (65).

#### Positive and negative affect

The Positive Affect and Negative Affect Schedule (PANAS) (66) (Dutch translation) (67) is a 20-item self-report questionnaire that measures the presence of certain affective states. The PANAS has two subscales: positive affect (PA) and negative affect (NA) that each consist out of 10 items. Participants had to rate the extent to which they experienced positive emotions (e.g., feeling “interested,” “enthusiastic,” “excited,” “proud,” “alert,” “active”) and negative emotions (e.g., feeling “guilty,” “pessimistic,” “distressed,” “upset,” “scared,” “irritable”) during the past 2 weeks on a 5-point Likert scale ranging from 1 (very slightly) to 5 (very much). The PANAS demonstrated good internal consistency for both the positive affect component (*α* = 0.85) and the negative component (*α* = 0.85). Previous research indicated good psychometric properties as well (67).

### Analytical approach

To investigate the effects of SMU reduction on mental well-being, this study used several approaches to analyze the data. After summarizing group characteristics and checking for significant between-group differences at baseline, we used MobileDNA data to check intervention adherence. Using Linear Mixed Modeling (LMM), the average social media use was compared across the three phases. That is, for each phase the daily time (in minutes) on social media was averaged across the two-week period, resulting in three repeated measures across participants. Using contrast and Holm-corrected *p*-values, differences between the average time using social media were compared between each phase, for the intervention and control group separately.

For our main analysis, Linear Mixed Modeling (LMM) was used to compare self-esteem, mindfulness, sleep and emotional well-being between the two groups (control vs. intervention) over time. The influence of the experimental manipulation (condition: control vs. SMU restriction) and time (baseline vs. post vs. follow up) was analyzed using LMM as implemented in the R packages lme4 (68) and lmerTest (69). The effect of phase was not modeled as a linear effect, but rather as a factor (i.e., categorical) with five levels and specific contrasts for pre-post differences were tested. We aggregated baseline data (phase one) from survey one and two, intervention data (phase two) from survey three and four, and used survey five for the follow-up data. The degrees of freedom in the analysis of deviance table were estimated using Satterthwaite’s method. As testing period 1 was a baseline measurement, any effect of intervention condition would show up as an interaction effect between time and condition for the variables.

### Additional analyses

To allow quantification of (strength of) evidence for the alternative versus null-hypothesis (70), we used Bayesian independent samples *t*-tests as implemented in JASP (version 0.18.3.0). The obtained Bayes Factors (BFs) provide an indicator of the likelihood of the observed data given the alternative hypothesis of beneficial effects of the intervention (BF10 > 1) versus the null hypothesis of no effects (BF01 > 1). These analyses were conducted on change scores reflecting potential improvement(s) on the variable(s) under investigation from baseline to the intervention phase. In line with prior analyses, we aggregated baseline data (survey 1 and 2) and intervention data (survey 3 and 4). Given the nature of our hypotheses, we relied on one-tailed tests. In line with Wagenmakers et al. (71, 72), we used following cut-offs for the BF: 1 = No evidence, 1–3 = Anecdotal evidence, 3–10 = Substantial evidence, 10–30 = Strong evidence, 30–100 = Very strong evidence, and >100 = Extreme evidence. We used the Cauchy distribution as prior and conducted robustness checks to evaluate the effects of prior choice. In addition, we investigated stability of the obtained BFs, exploring how evidence for the alternative versus null hypothesis cumulated as the sample size increased.

## Results

### Group characteristics

Demographic characteristics of participants by group can be found in Table 1. Independent-samples *t*-tests indicated no pre-existing differences for the main dependent variables between the two conditions except for one variable. Participants in the control condition scored significantly higher on the depression subscale from the DASS-21 compared to the experimental condition [*t*(62.89) = 2.50, *p* = 0.015]. In addition, a significant baseline difference was found between the participants in the experimental and control condition with respect to average screen time [*t*(65.97) = 2.37, *p* = 0.021]. At baseline, the average level of general screen time was higher in the control condition compared to the SMU reduction condition. With respect to the reported frequency of SMU and the use of communication applications, there were no significant differences between the two conditions at baseline (all *p*’s > 0.70).

**Table 1:** Demographic characteristics of participants by group.

Variable	Control (*N* = 32)	Experimental (*N* = 35)
Mean age (*SD*)	21.75 (1.97)	21.11 (1.45)
Gender		
*N* male (%)	5 (15.6)	4 (11.4)
*N* female (%)	26 (81.3)	31 (88.6)
*N* other (%)	1 (3.1)	0 (0)
Belgian nationality (%)	100	100

### Intervention adherence

The intervention’s effectiveness depended on how much participants adhered to the daily restrictions for social media applications. Data from the MobileDNA app allowed us to check for screen time in general, the amount they spent on apps to communicate with others (no restrictions) and the amount they spent on social media apps (30 min/day restriction). Table 2 shows the average screen times per usage type per period in each group.

**Table 2:** Summary of MobileDNA data for each type of smartphone usage in minutes.

Period	Control condition	Experimental condition
Mean and (*SD*) for screen time in general (min/day)		
Period 1 (baseline)	245 (74.6)	198 (86)
Period 2 (intervention)	238 (79.5)	129 (55.1)
Period 3 (follow-up)	276 (114)	198 (79.4)
Mean and (SD) for screen time SMU (min/day)		
Period 1 (baseline)	80 (41.2)	84.1 (48.8)
Period 2 (intervention)	80.9 (35.9)	19.8 (14.2)
Period 3 (follow-up)	103 (56.3)	79.6 (40.9)
Mean and (SD) for screen time communication (min/day)		
Period 1 (baseline)	45 (43)	47.8 (41.1)
Period 2 (intervention)	45.4 (43.8)	46.5 (30.9)
Period 3 (follow-up)	50.5 (57.1)	49.5 (54.8)

When looking at the average of social media usage for the three periods per condition, the contrast tests of the LMM with Holm corrected *p*-values showed that, as expected, there was no difference in average social media use in the control condition between the first and second period [*t*(112) = 1.047, *p* = 0.594]. There was some evidence that social media use in the third period increased with about 21 min compared to the first period [*t*(112) = 3.636, *p* = 0.002]. In the experimental group, average social media use decreased due to the intervention with about 1 h in the second period compared to the first phase [*t*(114) = −10.985, *p* < 0.0001]. However, when the intervention was stopped, the average social media use recovered completely with an increase of about 1h between the second and third period. In addition, there was no evidence for a difference in social media use in the intervention group between the first and third period [*t*(115) = −0.597, *p* = 0.59], suggesting that there was no overcompensation or rebound in social media use due to “lost time online” during the intervention.

In addition to social media use, we also looked at the amount of time spent online communicating with others. Table 2 summarizes the group levels for mean screen time on communication applications (e.g., WhatsApp, Messenger). Using the same combination as we did with social media use across the different periods, we found no evidence for differences in time spent online communicating (all Holm corrected *p*’s = 1).

In line with this, we observed that 25 of the 35 participants in the experimental group fully adhered to the SMU restriction (max. 30 min/day). Four participants did not completely follow the restriction and showed a medium adherence (30-45 min/day). For the remaining six participants, we were not able to evaluate the intervention adherence due to technical issues in the smartphone monitoring application.^1^ In conclusion, although there was variability in adherence to the intervention, we still observed the intervention’s impact on screen time at the between-subject level.

### Main analysis

**Figure 3:**
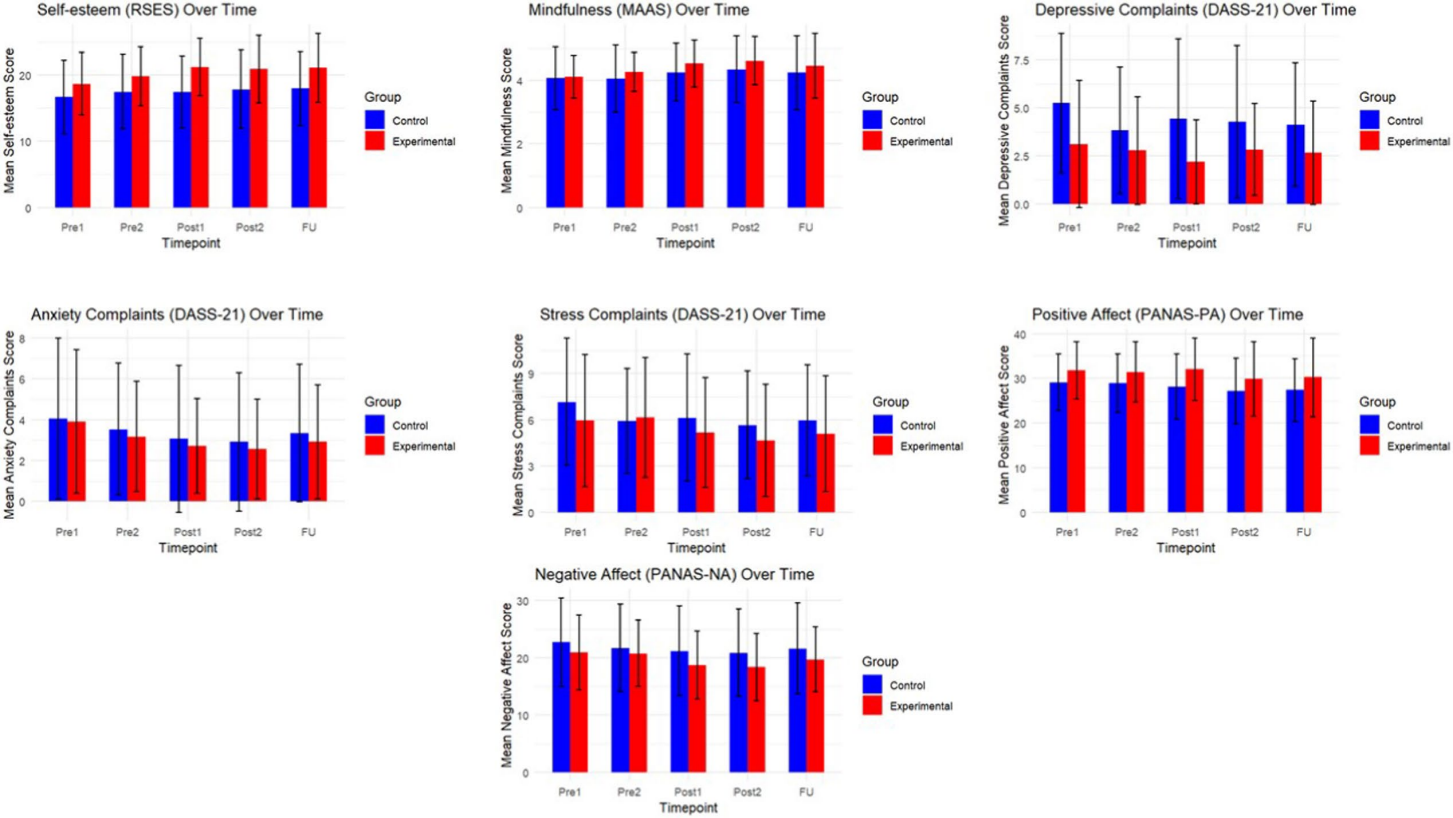
Graphical representation of the self-reported outcomes over time.

**Figure 4:**
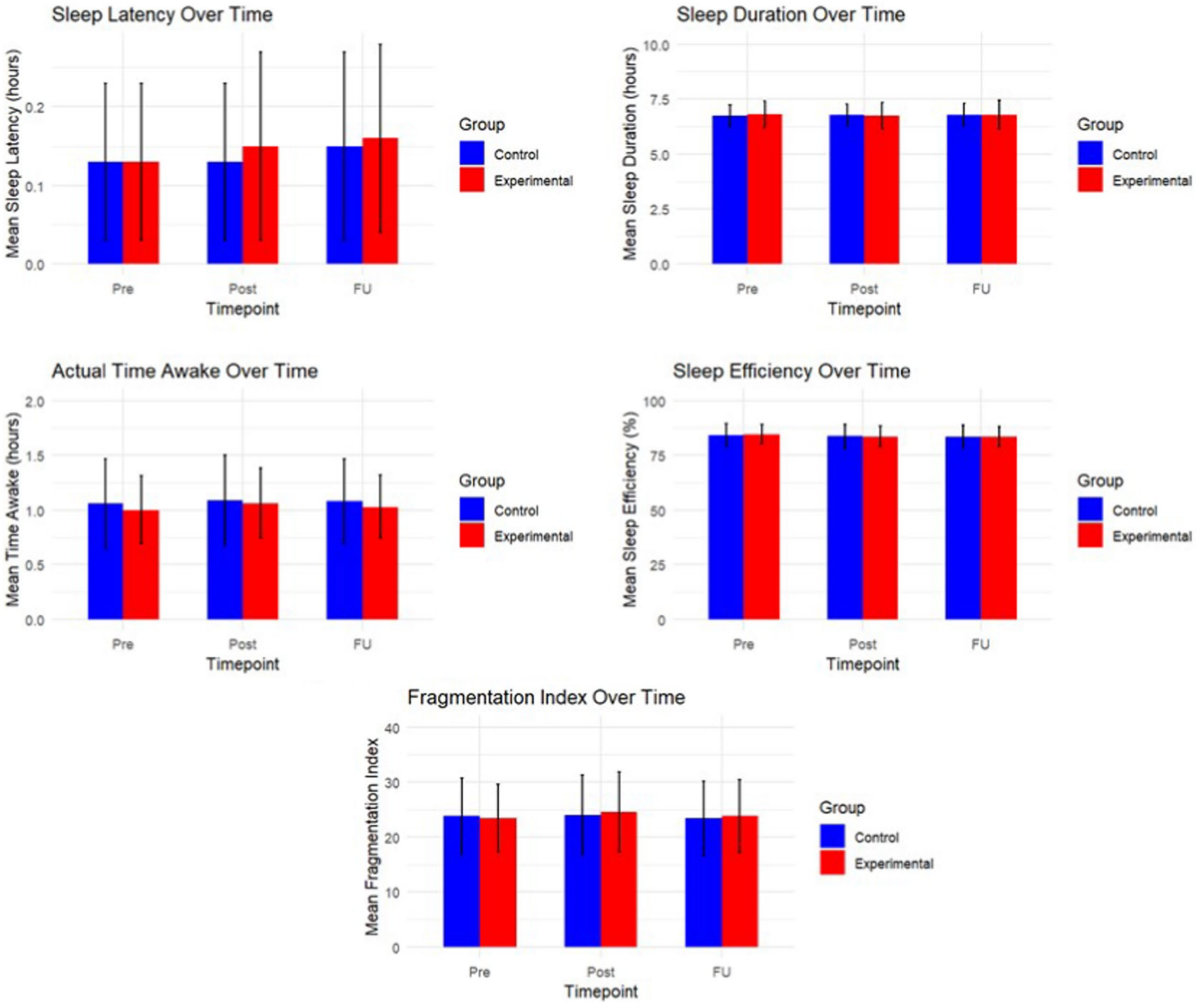
Graphical representation of the objective sleep outcomes over time.

The mean values of self-esteem, mindfulness, the internalizing complaints, positive affect and negative affect at the five testing periods are summarized in Table 3 (see Figure 3 for a graphical representation). The mean values of the different sleep outcomes can be consulted in Table 4 (see Figure 4 for a graphical representation). The corresponding statistics from our analyses are represented in Table 5.

**Table 3:** Means and standard deviations (SD) for the self-reported outcomes.

Condition	Pre1	Pre2	Post1	Post2	FU	TOTAL
Self-esteem: RSES						
Con	16.69 (5.56)	17.44 (5.65)	17.44 (5.41)	17.84 (5.93)	17.94 (5.56)	17.47 (5.41)
Exp	18.66 (4.72)	19.80 (4.47)	21.20 (4.30)	20.89 (5.10)	21.06 (5.21)	20.32 (4.36)
TOTAL	17.72 (5.19)	18.67 (5.17)	19.40 (5.18)	19.43 (5.68)	19.55 (5.57)	
Mindfulness: MAAS						
Con	4.07 (0.99)	4.06 (1.06)	4.25 (0.91)	4.35 (1.04)	4.24 (1.15)	4.19 (0.91)
Exp	4.11 (0.67)	4.27 (0.62)	4.53 (0.74)	4.62 (0.75)	4.46 (1.02)	4.40 (0.62)
TOTAL	4.09 (0.83)	4.17 (0.86)	4.40 (0.83)	4.49 (0.90)	4.36 (1.08)	
Depressive complaints: DASS-21 (dep)						
Con	5.25 (3.64)	3.84 (3.31)	4.44 (4.17)	4.28 (3.97)	4.13 (3.23)	4.39 (2.92)
Exp	3.11 (3.31)	2.77 (2.80)	2.17 (2.19)	2.83 (2.39)	2.65 (2.70)	2.70 (2.22)
TOTAL	4.13 (3.61)	3.28 (3.08)	3.25 (3.46)	3.52 (3.30)	3.36 (3.04)	
Anxiety complaints: DASS-21 (anx)						
Con	4.06 (3.94)	3.53 (3.24)	3.06 (3.59)	2.91 (3.38)	3.34 (3.37)	3.38 (3.00)
Exp	3.91 (3.53)	3.17 (2.70)	2.71 (2.33)	2.57 (2.45)	2.91 (2.78)	3.07 (2.13)
TOTAL	3.99 (3.70)	3.34 (2.95)	2.88 (2.98)	2.73 (2.92)	3.12 (3.06)	
Stress complaints: DASS-21 (str)						
Con	7.16 (4.14)	5.91 (3.40)	6.13 (4.13)	5.66 (3.49)	5.97 (3.60)	6.16 (3.04)
Exp	5.94 (4.27)	6.14 (3.89)	5.17 (3.56)	4.66 (3.64)	5.09 (3.74)	5.39 (3.18)
TOTAL	6.52 (4.22)	6.03 (3.64)	5.63 (3.84)	5.13 (3.58)	5.52 (3.62)	
Positive affect: PANAS-PA						
Con	29.09 (6.33)	28.88 (6.54)	28.13 (7.28)	27.13 (7.43)	27.34 (7.05)	28.11 (6.15)
Exp	31.74 (6.41)	31.43 (6.73)	32.00 (6.95)	29.86 (8.32)	30.24 (8.80)	31.08 (6.30)
TOTAL	30.48 (6.46)	30.21 (6.71)	30.15 (7.32)	28.55 (7.97)	28.83 (8.07)	
Negative affect: PANAS-NA						
Con	22.75 (7.75)	21.75 (7.64)	21.25 (7.87)	20.91 (7.60)	21.66 (7.94)	21.66 (6.66)
Exp	20.97 (6.54)	20.80 (5.80)	18.71 (5.98)	18.40 (5.93)	19.74 (5.71)	19.75 (4.75)
TOTAL	21.82 (7.14)	21.25 (6.70)	19.93 (7.01)	19.60 (6.84)	20.67 (6.90)	

**Table 4:** Means and standard deviations (SD) of sleep outcomes.

Condition	Pre	Post	FU	TOTAL
Sleep latency (in hours)				
Con	0.13 (0.1)	0.13 (0.1)	0.15 (0.12)	0.13 (0.1)
Exp	0.13 (0.1)	0.15 (0.12)	0.16 (0.12)	0.14 (0.11)
TOTAL	0.13 (0.1)	0.14 (0.11)	0.15 (0.12)	
Sleep duration (in hours)				
Con	6.75 (0.49)	6.78 (0.5)	6.79 (0.52)	6.77 (0.5)
Exp	6.81 (0.59)	6.75 (0.59)	6.79 (0.65)	6.78 (0.61)
TOTAL	6.78 (0.54)	6.77 (0.55)	6.79 (0.59)	
Actual time awake (in hours)				
Con	1.06 (0.41)	1.09 (0.41)	1.08 (0.39)	1.07 (0.4)
Exp	1.00 (0.31)	1.06 (0.32)	1.03 (0.29)	1.03 (0.3)
TOTAL	1.02 (0.36)	1.07 (0.36)	1.05 (0.34)	
Sleep efficiency (in percentage)				
Con	84.09 (5.16)	83.72 (5.5)	83.56 (5.04)	83.79 (5.18)
Exp	84.64 (4.31)	83.64 (4.8)	83.56 (4.54)	83.95 (4.53)
TOTAL	84.39 (4.69)	83.67 (5.1)	83.56 (4.74)	
Fragmentation index (in percentage)				
Con	23.83 (6.88)	24.06 (7.18)	23.43 (6.77)	23.77 (6.87)
Exp	23.48 (6.14)	24.59 (7.28)	23.85 (6.62)	23.97 (6.64)
TOTAL	23.64 (6.44)	24.34 (7.17)	23.64 (6.64)	

**Table 5:** Summary the main and interaction effects.

Outcome	Main effects time	Main effects condition	Interaction effects (t*c)
Main outcomes:			
RSES	*F*(4, 258.03) = 10.3, *p* < 0.001^***^	*F*(1, 65.00) = 5.7, *p* = 0.020^*^	*F*(4, 258.03) = 2.2, *p* = 0.073
MIND	*F*(4, 258.12) = 6.3, *p* < 0.001^***^	*F*(1, 65.03) = 1.2, *p* = 0.28	*F*(4, 258.12) = 0.6, *p* = 0.698
DASS dep	*F*(4, 258.25) = 2.0, *p* = 0.097	*F*(1, 65.12) = 7.2, *p* = 0.009^**^	*F*(4, 258.25) = 0.9, *p* = 0.467
DASS anx	*F*(4, 258.13) = 3.9, *p* = 0.005^**^	*F*(1, 65.01) = 0.3, *p* = 0.61	*F*(4, 258.13) = 0.0, *p* = 0.997
DASS str	*F*(4, 258.18) = 3.2, *p* = 0.014^*^	*F*(1, 65.06) = 1.0, *p* = 0.317	*F*(4, 258.18) = 0.9, *p* = 0.449
PANAS-PA	*F*(4, 258.04) = 3.0, *p = 0*.020^*^	*F*(1, 64.95) = 3.8, *p* = 0.057	*F*(4, 258.04) = 0.3, *p* = 0.879
PANAS-NA	*F*(4, 258.18) = 3.1, *p* = 0.016^*^	*F*(1, 65.07) = 1.9, *p* = 0.169	*F*(4, 258.18) = 0.3, *p* = 0.873
Sleep outcomes:			
Latency	*F*(2, 118.46) = 2.54, *p* = 0.083	*F*(1, 59.97) = 0.16, *p* = 0.694	*F*(2, 118.46) = 0.51, *p* = 0.604
Duration	*F*(2, 118.43) = 0.09, *p* = 0.914	*F*(1, 60.09) = 0.01, *p* = 0.90	*F*(2, 118.43) = 0.35, *p* = 0.706
Time awake	*F*(2, 118.22) = 2.79, *p* = 0.065	*F*(1, 60.09) = 0.26, *p* = 0.61	*F*(2, 118.22) = 0.40, *p* = 0.67
Efficiency	*F*(2, 118.29) = 3.59, *p* = 0.031^*^	*F*(1, 60.12) = 0.02, *p* = 0.879	*F*(2, 118.29) = 0.52, *p* = 0.596
Frag. index	*F*(2, 118.32) = 1.44, *p* = 0.242	*F*(1, 60.13) = 0.02, *p* = 0.884	*F*(2, 118.32) = 0.58, *p* = 0.561

^*^p < 0.05, ^**^p < 0.01, ^***^p < 0.001.

Given that this study aimed to evaluate whether the intervention led to changes over time, our primary hypothesis focused on the interaction between time and condition. Across all main outcomes, we found no significant interaction effects, indicating that the pattern of change over time did not differ between the experimental and control groups (all *p* > 0.07; see Table 5). These findings suggest that the intervention did not produce immediate or consistent effects beyond natural changes that occurred across time in both groups.

Although several outcomes showed significant main effects of time, these effects were present in both groups and are therefore not attributable to the intervention. Specifically, we observed time-related changes in self-esteem [*F*(4, 258.03) = 10.3, *p* < 0.001], mindfulness [*F*(4, 258.12) = 6.3, *p* < 0.001], anxiety [*F*(4, 258.13) = 3.9, *p* = 0.005], stress [*F*(4, 258.18) = 3.2, *p* = 0.014], positive affect [*F*(4, 258.04) = 3.0, *p* = 0.020], negative affect [*F*(4, 258.18) = 3.1, *p* = 0.016], and sleep efficiency [*F*(2, 118.29) = 3.59, *p* = 0.031].

In terms of main effects of condition, self-esteem [*F*(1, 65.00) = 5.7, *p* = 0.020] and depressive complaints [*F*(1, 65.12) = 7.2, *p* = 0.009] differed between groups overall, However, given the absence of interaction effects, these differences likely reflect baseline variation or other non-specific factors, rather than a systematic effect of the intervention itself.

### Additional analyses

#### Bayesian analysis

We conducted follow-up Bayesian analyses to assess how likely the observed pattern of results was under the assumption that the intervention had immediate effects (alternative hypothesis, BF10 > 1) compared to no such effects (null hypothesis, BF01 > 1). Except for self-esteem, for which substantial evidence was obtained favoring the alternative hypothesis of beneficial effects of the intervention (BF10 = 4.10), evidence for most other variables favored the null-hypothesis or was inconclusive (e.g., mindfulness: BF10 = 1.12). For example, for anxiety complaints, the results indicated that the data were 3.53 times more likely under the assumption that the intervention had no effect than under the assumption that it did (BF01 = 3.53). Similarly, substantial evidence favoring the null hypothesis was obtained for sleep latency (BF01 = 6.50), sleep duration (BF01 = 6.20), actual time awake (BF01 = 6.55), sleep efficiency (BF01 = 6.62), and fragmentation index (BF01 = 6.68). For stress complaints (BF01 = 1.92), depressive complaints (BF01 = 2.77), positive affect (BF01 = 2.28) and negative affect (BF01 = 1.61), the evidence was weaker but still leaned toward supporting no effect. For sequential analyses and investigation of effects of the prior used (see Supplementary Figures 1–12).

## Discussion

The current study applied a longitudinal experimental design to examine the causal influence of SMU on different mental well-being parameters, both subjectively and objectively monitored. This approach addressed the limitations of prior research, which has largely relied on cross-sectional designs to investigate the associations between SMU and mental well-being (73). The aim of this study was to investigate the effects of reducing SMU intensity on psychological measures of self-esteem, mindfulness, sleep, and emotional well-being in young adults with high social media use. We particularly chose to focus on high social media users, as previous literature showed that disruptions in mental well-being are more likely to occur in high frequent social media users (47–49). To compensate for the shortcomings in previous literature, we monitored SMU, included a wider variety of mental well-being outcomes and added a behavioral approach to supplement findings with objectively recorded sleep data.

Despite strong claims about the effects of SMU on mental well-being [e.g., (46)], results indicate that the SMU intervention did not lead to any significant improvements of self-esteem, mindfulness, sleep or emotional well-being, compared to using social media as usual. These findings add to recent literature (18, 45, 74) that has failed to observe strong effects of SMU on mental well-being, but considering this is a null finding we first discuss the validity of this null finding. One explanation for these null findings is related to statistical power to detect effects in our study. Based on a sensitivity analysis using G*Power3 (75) for between-within interaction effects with five measurements and two groups, we calculated that our study had a sufficiently big sample to detect an effect size of *f* = 0.15 with 1 − *β* = 0.80, which represents a small (to medium) effect. For correlations of 0.5 between the repeated measures (which corresponds to our sample), we would need 56 participants to get a power > 0.8. In order to detect even smaller effect sizes (*f* = 0.10) we would have needed 122 participants to obtain the same power (1 − β = 0.80). This study could not reach the required number of participants to observe small effect sizes given the limited number of available wearables to collect physiological data on sleep. Based on this power analysis, if the true effects were of medium to large size (*f* ≥ 0.15), our study should have been able to observe them. If there are effects of a social media reduction on mental well-being, they are likely to be small, even though our study did not find any. In line with this, follow-up Bayesian analyses indicate that for most outcomes, cumulative evidence was in favor of the null hypothesis of no immediate beneficial effects of SMU reduction on mental well-being.

A second explanation for the absence of any intervention effect might be because we decided to go for a restriction of use instead of having participants fully stop any SMU. We opted for this experimental condition since it was deemed more feasible and ecologically valid to implement rather than stopping SMU completely. Moreover, as expected, this approach led to less selective drop-out compared to complete abstinence studies (45). There are various possible explanations why this more flexible approach resulted in null findings. First, research is beginning to show that it is not screen time on social media as such, but rather experiences while using it that are more critical to well-being. For example, a recent study found that the detrimental effects of objectively monitored screen time on social media on affective well-being and goal interference are mitigated when the use of social media is fulfilling adolescents’ basic psychological needs (76). As such, it might be that the present reduction in SMU only led to reductions in screen time, but did not alter the amount of need fulfillment, thereby not yielding any subsequent changes in terms of the mental well-being outcomes. Next to need fulfillment, there are various other potential pathways through which SMU might influence mental well-being, such as social connections (77) or lifestyle behaviors (78). If these factors remained unchanged during the intervention, then reducing SMU alone would not be expected to lead to improvements. This supports the notion that interventions targeting SMU in isolation may be insufficient to enhance mental well-being, and that a broader focus on the psychosocial environment might be necessary. Alternatively, it is noteworthy that at the group level, this condition led to a large decrease in SMU. However, in this condition, not all participants strictly adhered to the maximum 30 min per day. To clearly see causal effects, a restriction intervention might be too lenient and total abstinence could be required. This allows for complete disconnection and avoids the temptation to go on social media (39, 79).

The current results add to the mixed findings in this literature (18, 45). Interestingly, several initial studies found rather large effects of restricting SMU on mental health outcomes (38, 80). However, when applying a more rigorous methodological approach to these types of experimental studies (e.g., handling demand characteristics), research failed to replicate such effects and mainly found no differences between a restriction versus a SMU as usual condition (45). Our study set out to include a wider array of mental well-being outcomes, whereas previous studies mainly focused on more distal variables such as depression and anxiety (38).

The current study has several drawbacks that are important to mention. First, considering our participants, this study has a rather homogeneous sample where all participants were university students. This complicates drawing inferences about the effects of reduced SMU on mental well-being of the general population. However, this group is particularly important to involve in this kind of research, provided that adults under 29 years old are especially likely to report SMU (81). Alternately, there were some baseline differences between groups (for depressive complaints and general screen time) that may confound between-group comparisons. Although such differences are not unexpected given the number of outcome variables, they are to be taken into account when making interpretations. However, our primary analysis focused on the time x condition interactions, which were non-significant across all outcomes. This suggest that the lack of significant interactions cannot be attributed solely to baseline differences. Second, the timeline in our study was based on practical constraints of the MotionWatch. Since it needed periods of 2 weeks to draw proper conclusions on participants’ sleep, each phase (including the intervention) consisted of 2 weeks. Given that we were not able to find any significant results of the intervention over time, the possibility exists that the intervention period was not long enough for the effects of a SMU reduction to be observed. Future studies could take this into account by extending the duration of the reduction. Third, this study aimed to recruit participants who reported a high frequency of SMU (>2 h/day) given that these participants are most likely to benefit from reduced SMU (47–49). Our data shows that the participants included in this study used social media on average for 1.5 h each day in general. This might be due to an overestimation of their own SMU based on their total screen time which was on average almost 4 h on a daily basis. An alternative explanation is the Hawthorne effect, where participants’ awareness of their SMU being monitored may have resulted in changes to their SMU behavior (82). To this end, we tried to minimize the impact of this effect by disabling the option to receive feedback in the MobileDNA application regarding their own SMU. This, however, does not eliminate the notion that participants were aware of the continuous monitoring. Finally, this study was not pre-registered according to open-science principles. We believe that future research in this field should adhere to open-science principles as much as possible. It is noteworthy that the dataset of this study has been made publicly available on the Open Science Framework^2^ to increase transparency.

This study contributes to the growing knowledge about the impact of SMU on young adults’ mental well-being. The aim was to investigate whether reducing the intensity of SMU has an effect on self-esteem, mindfulness, sleep, internalizing complaints, positive and negative affect using a well-controlled longitudinal design that manipulated SMU over time within the same participants. In the end, no significant findings were found after reducing SMU, suggesting that future studies should focus more on the motivation and content of SMU rather than absolute intensity of use. Additionally, future studies could consider incorporating healthy emotion coping strategies to optimize the SMU restriction intervention.

## Data Availability

The datasets presented in this study can be found in online repositories. The names of the repository/repositories and accession number(s) can be found at: OSF, https://osf.io/cqyt8.

## Footnotes

^1^Re-analyzing the data excluding these individuals did not impact our main pattern of findings.

^2^ https://osf.io/cqyt8
