# Supplementary material for: Causal effects of social media use on self-esteem, mindfulness, sleep and emotional well-being: a social media restriction study
Source: Front Public Health. 2025 May 30;13:1548504. doi: 10.3389/fpubh.2025.1548504 (PMC12162677; doi:10.3389/fpubh.2025.1548504)
Supplement: Supplementary file 1 [file Supplementary_file_1.docx]

**Supplemental materials**


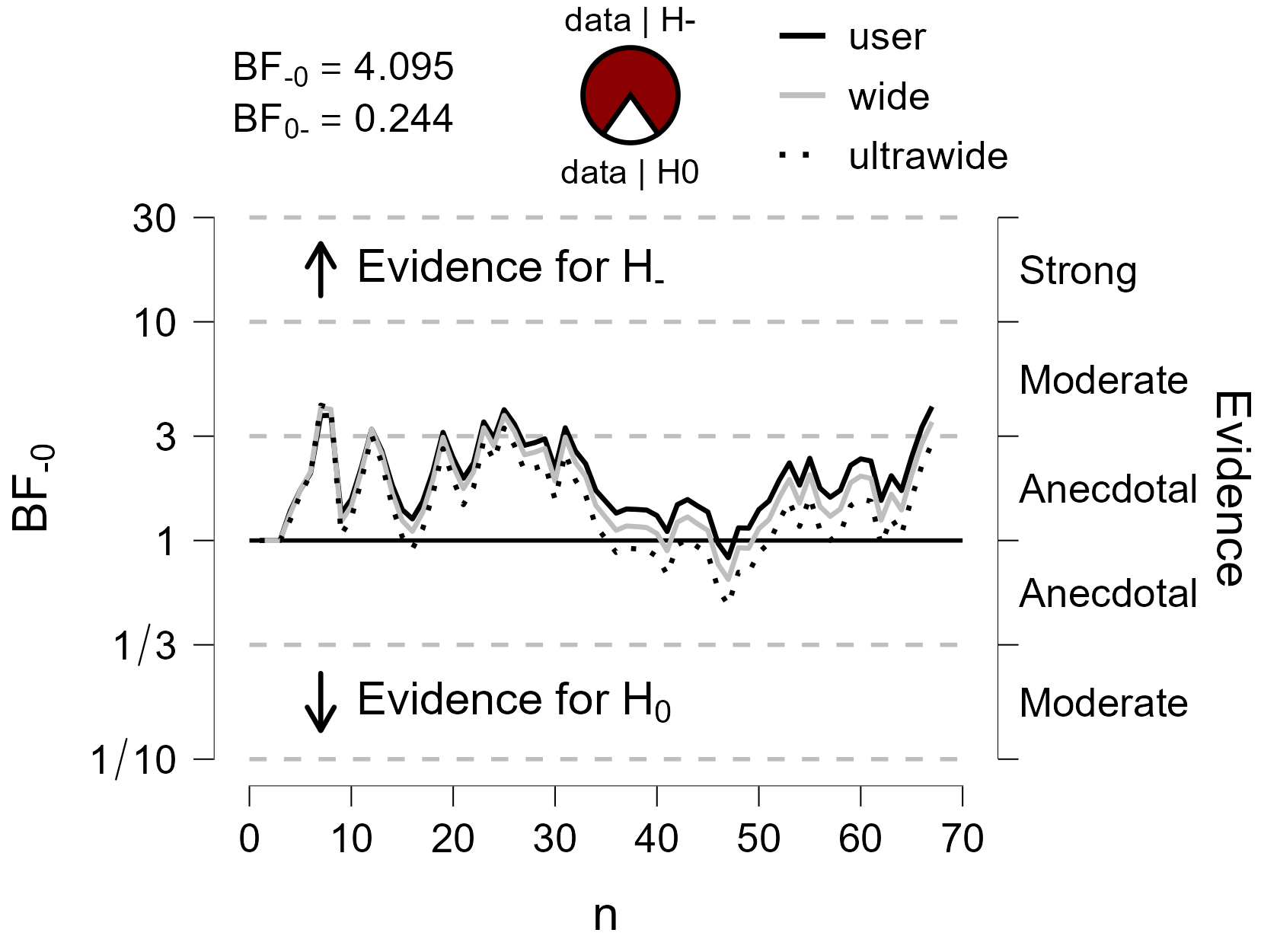


*Supplemental Figure 1. Cumulative evidence for the alternative vs. null-hypothesis of (no) beneficial effects on SMU reduction on self-esteem.
Note:* This plot presents the results of sequential analyses (Bayesian Independent samples *t*-tests) on change scores for self-esteem, representing how evidence for one hypothesis over the other cumulates as the sample-size increased. A Cauchy prior has been implemented. Lines represent the impact of prior choice on strength of evidence.


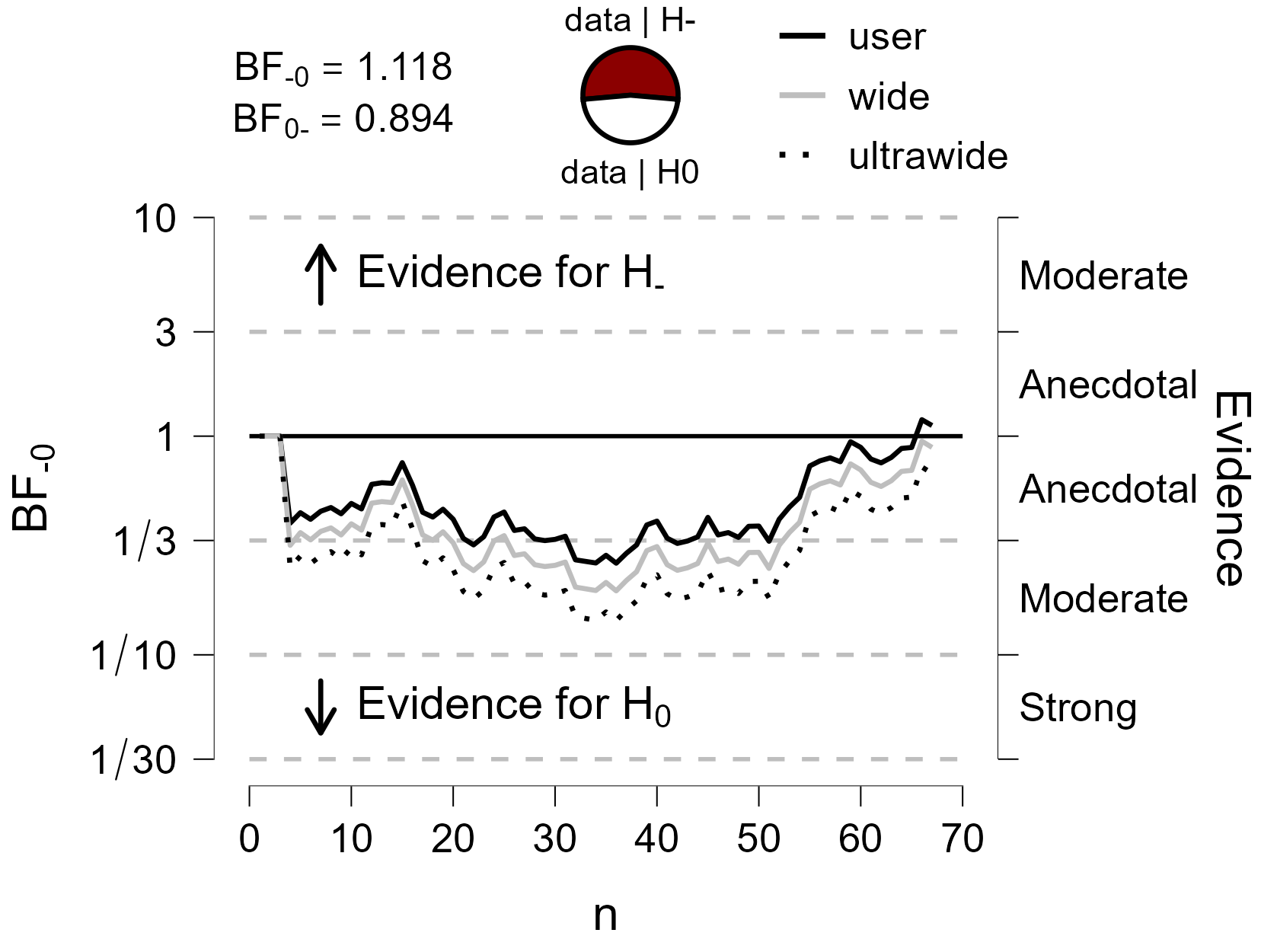


*Supplemental Figure 2. Cumulative evidence for the alternative vs. null-hypothesis of (no) beneficial effects on SMU reduction on mindfulness.*


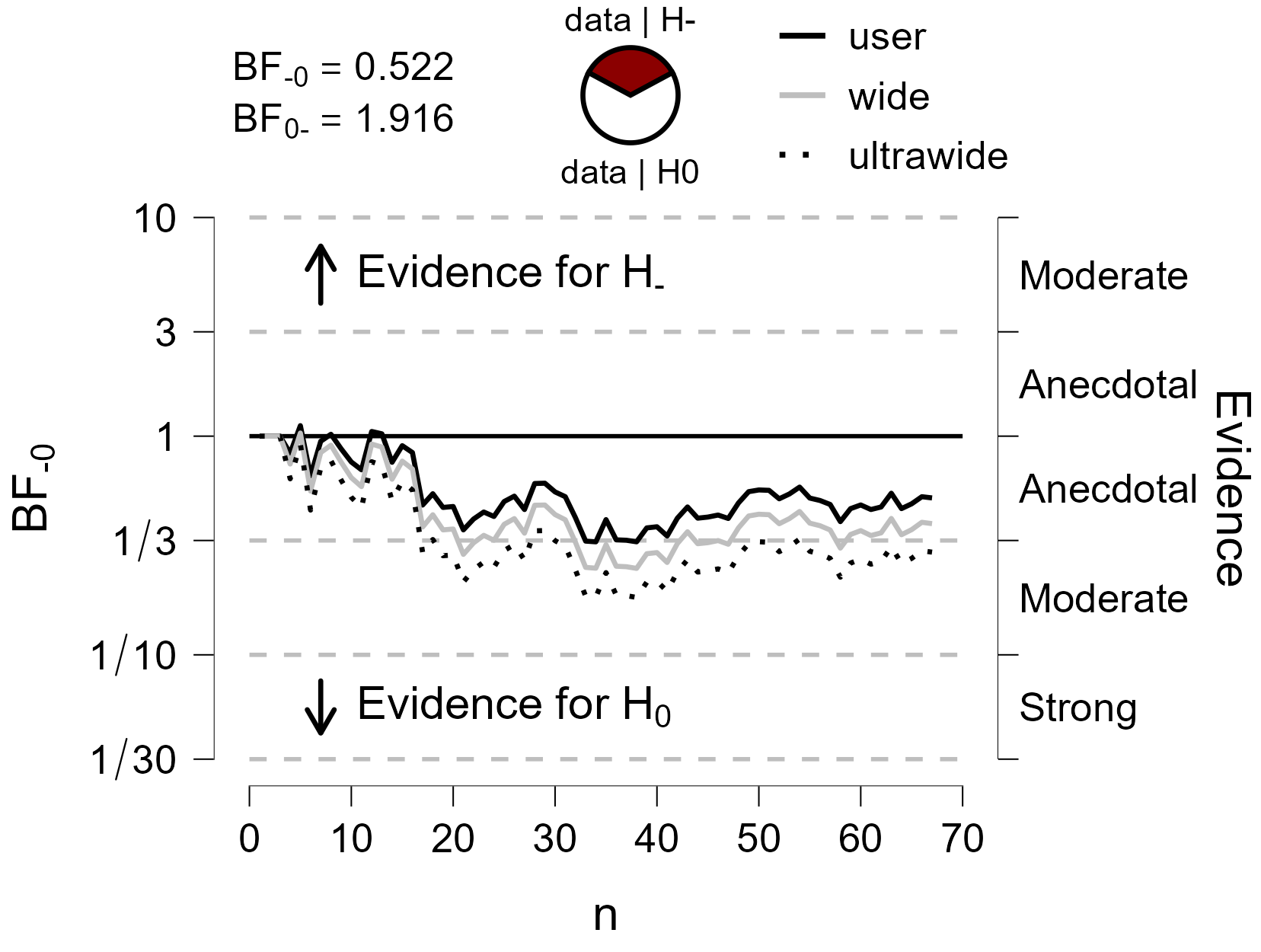


*Supplemental Figure 3. Cumulative evidence for the alternative vs. null-hypothesis of (no) beneficial effects on SMU reduction on stress complaints.*


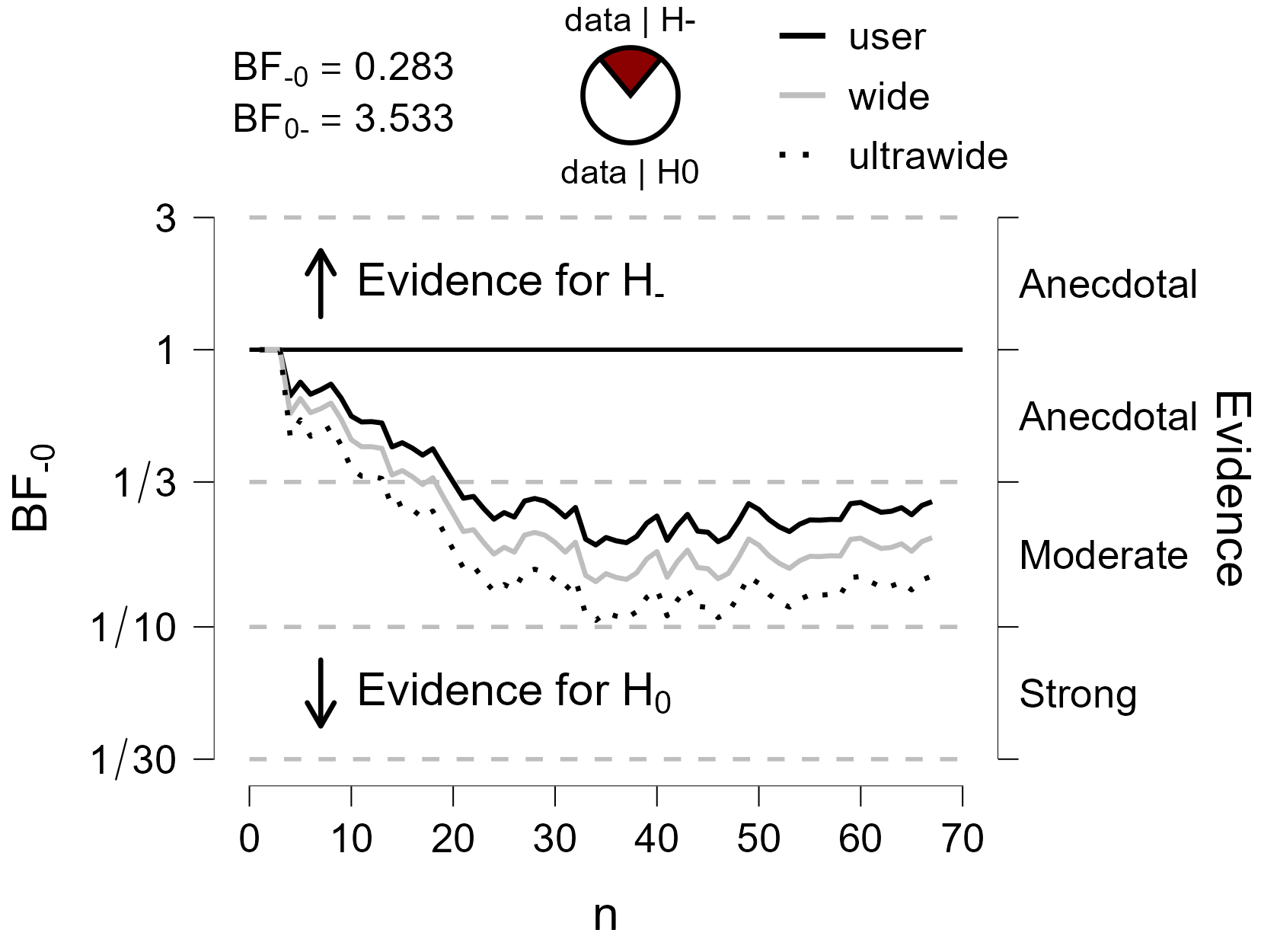


*Supplemental Figure 4. Cumulative evidence for the alternative vs. null-hypothesis of (no) beneficial effects on SMU reduction on anxiety complaints.*


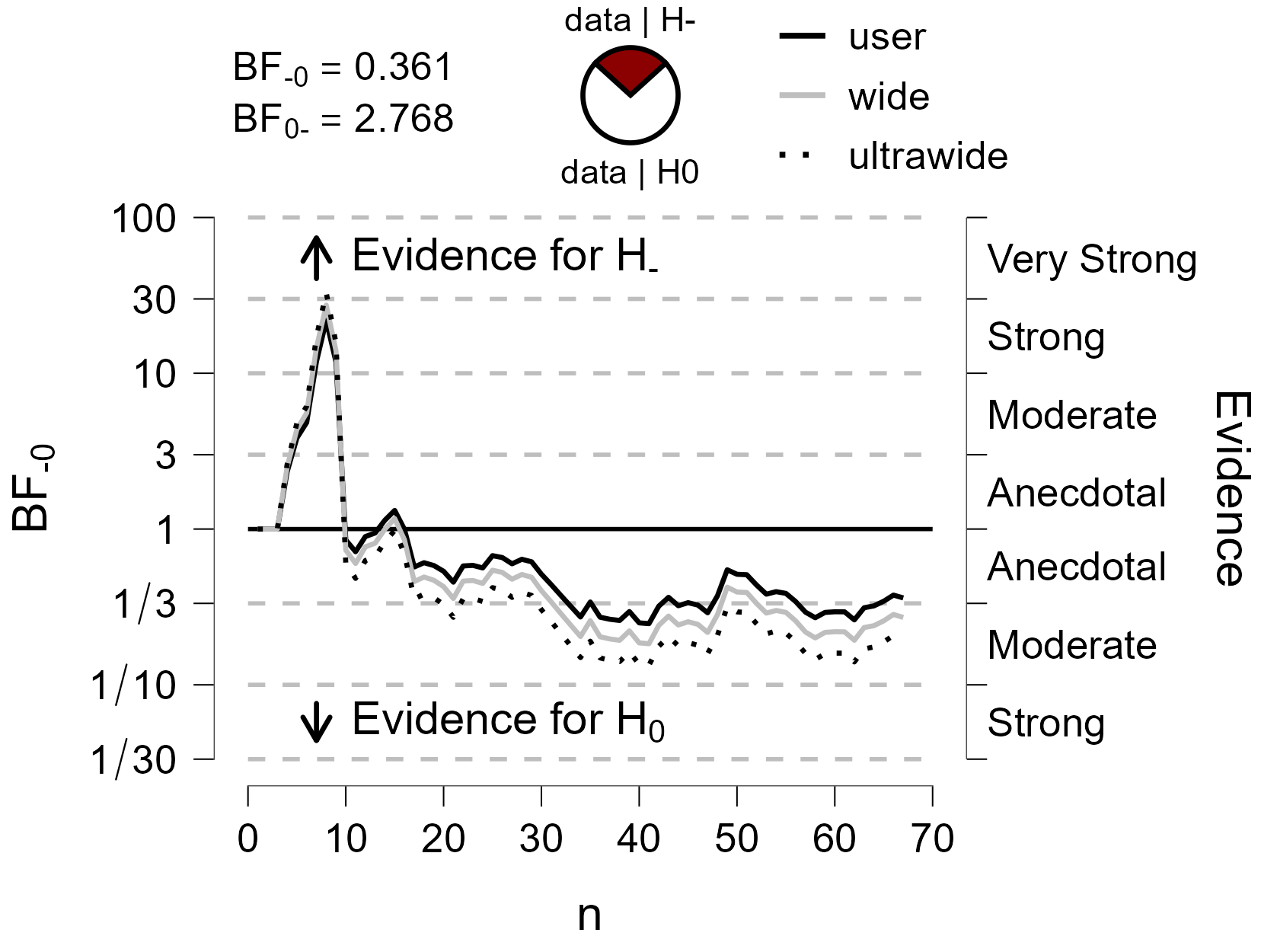


*Supplemental Figure 5. Cumulative evidence for the alternative vs. null-hypothesis of (no) beneficial effects on SMU reduction on depressive complaints.*

*
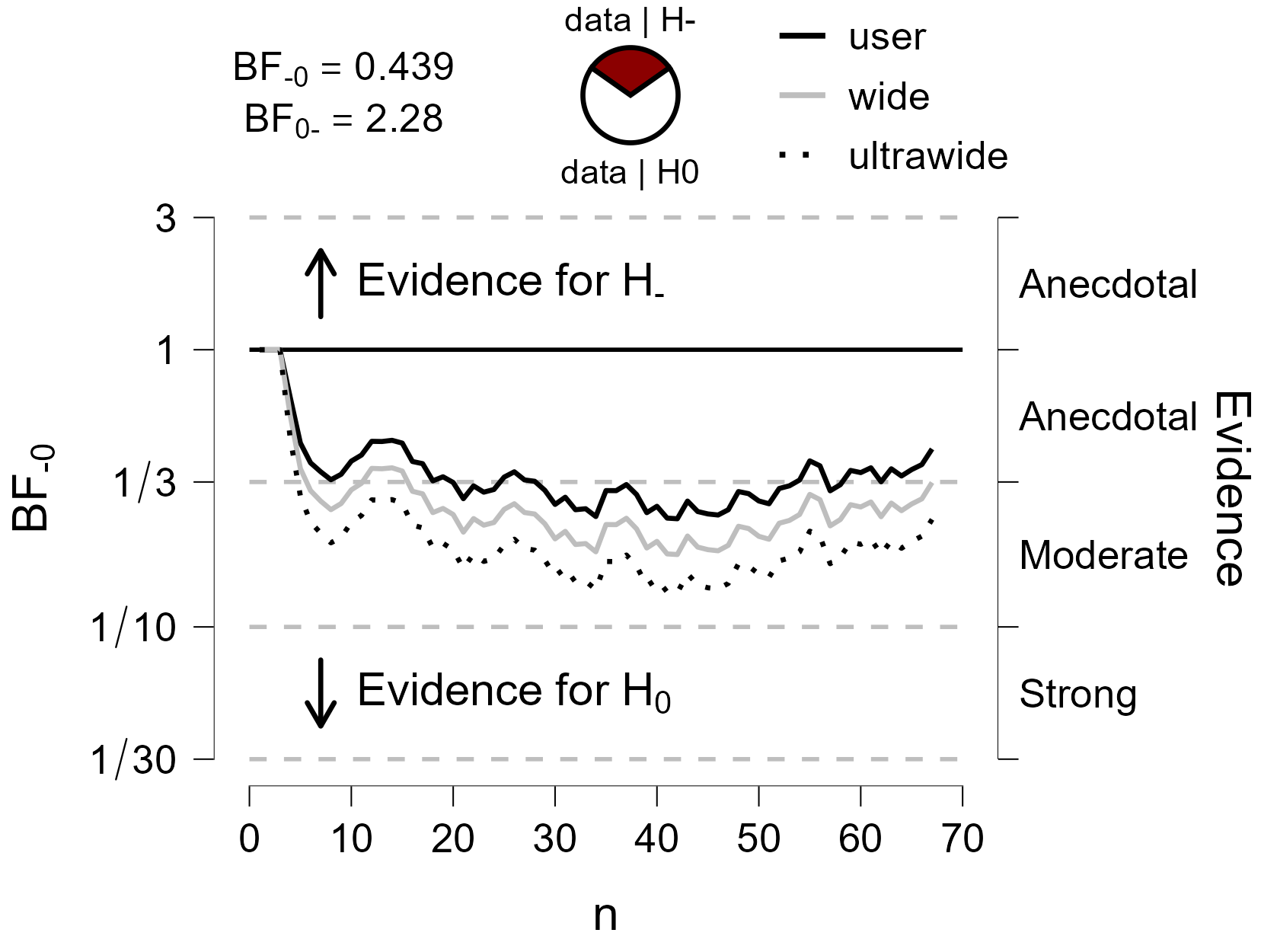
*

*Supplemental Figure 6. Cumulative evidence for the alternative vs. null-hypothesis of (no) beneficial effects on SMU reduction on positive affect.*

*
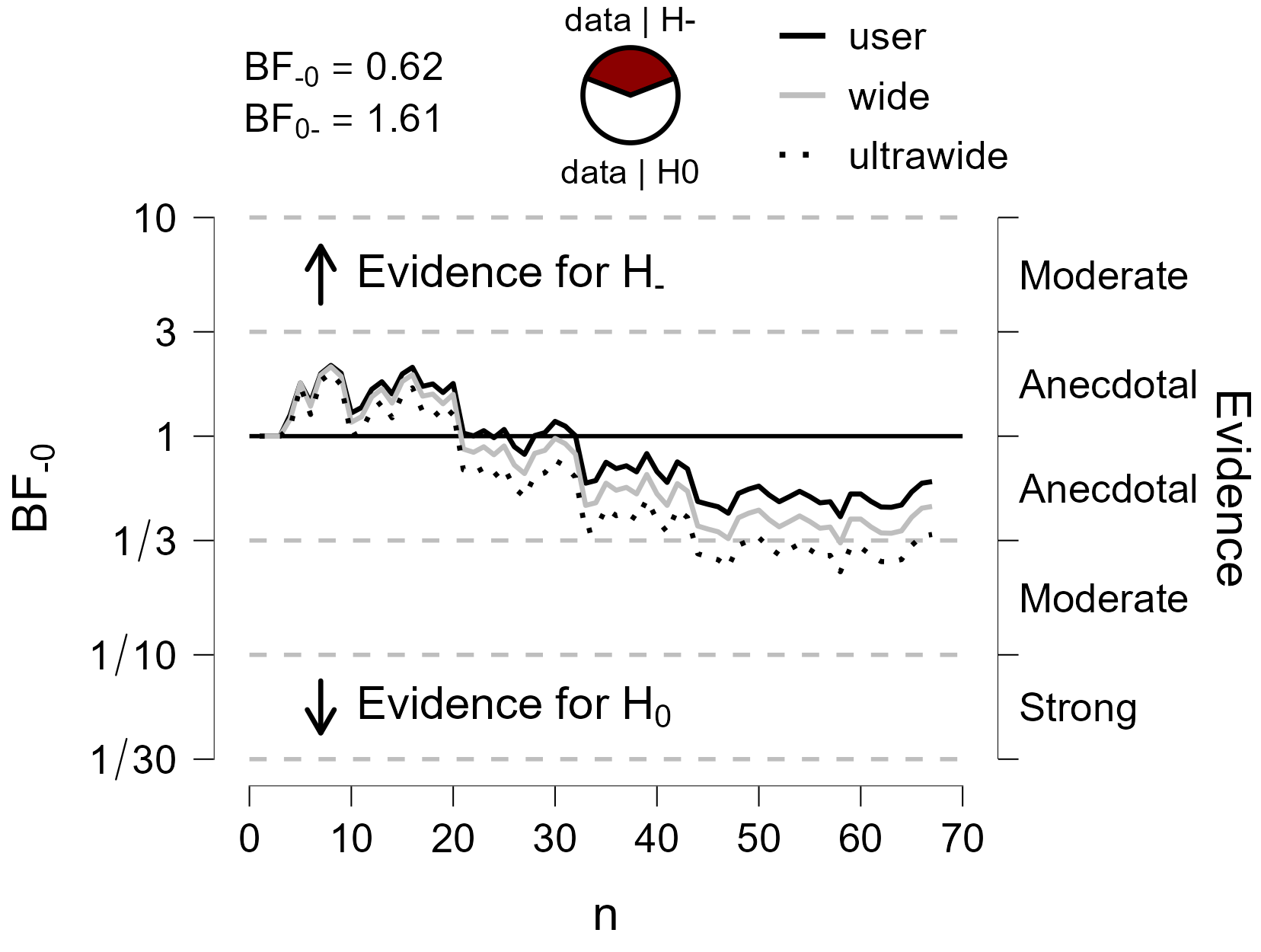
*

*Supplemental Figure 7. Cumulative evidence for the alternative vs. null-hypothesis of (no) beneficial effects on SMU reduction on negative affect.*

*
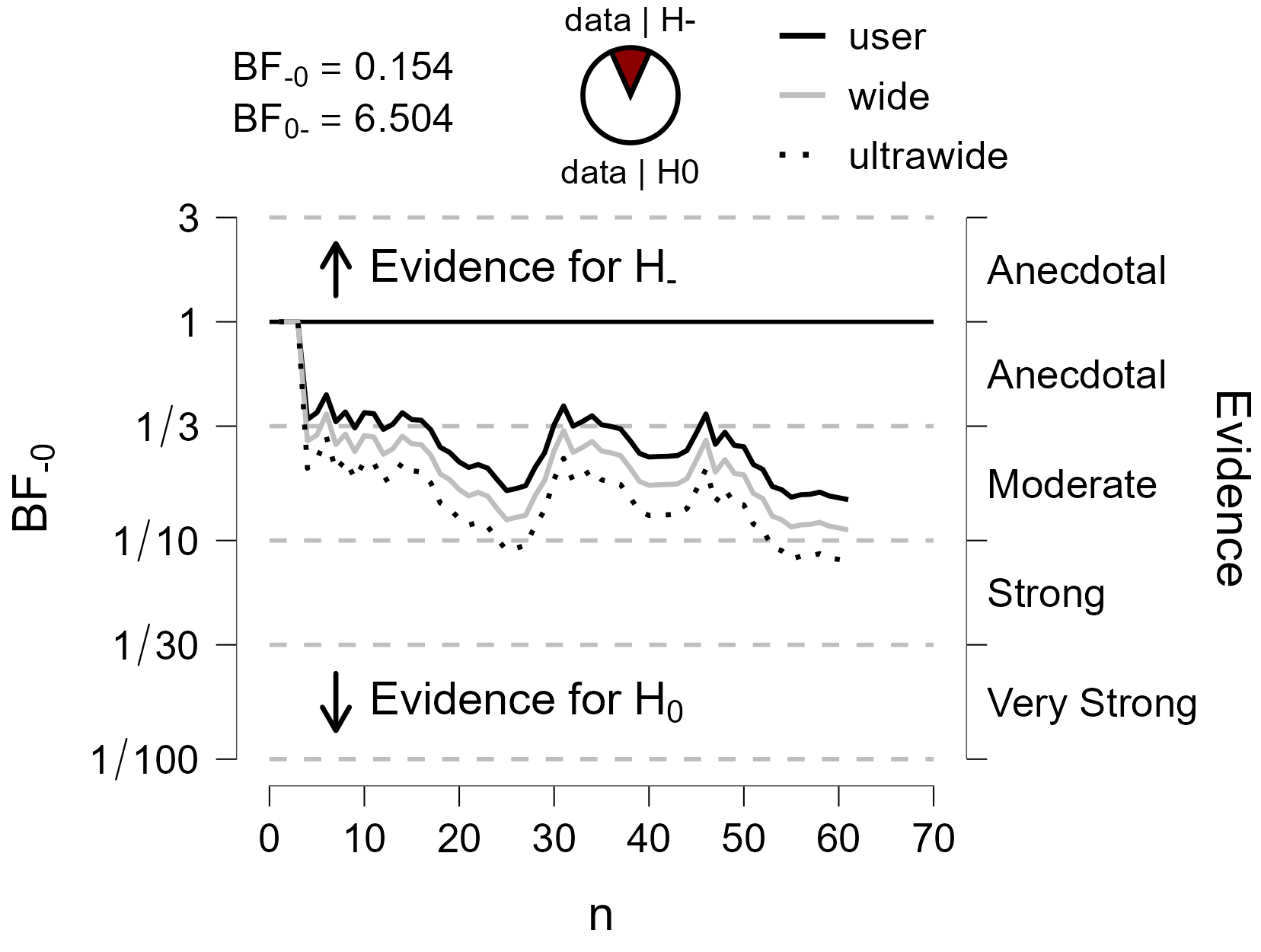
*

*Supplemental Figure 8. Cumulative evidence for the alternative vs. null-hypothesis of (no) beneficial effects on SMU reduction on sleep latency.*

*
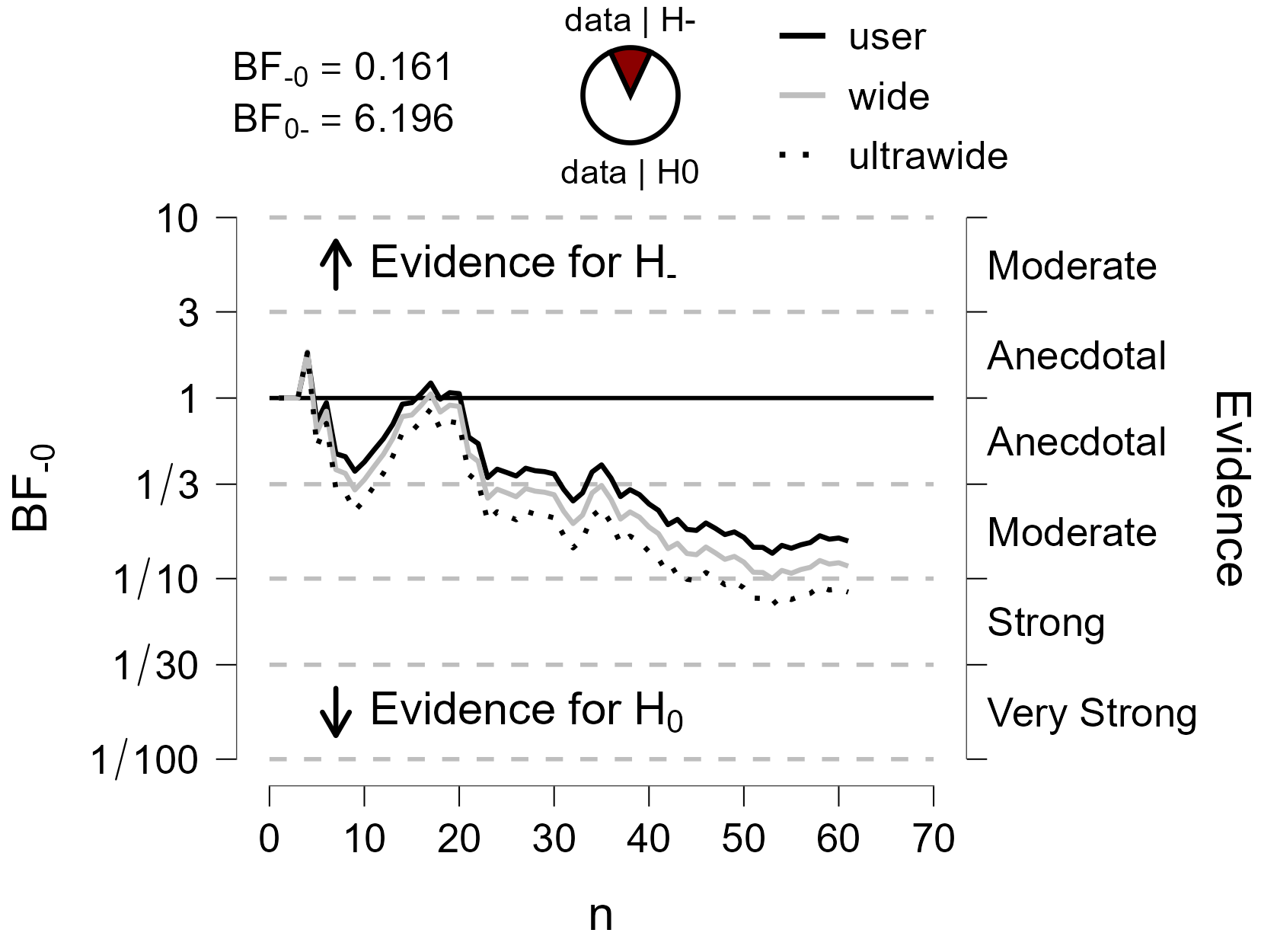
*

*Supplemental Figure 9. Cumulative evidence for the alternative vs. null-hypothesis of (no) beneficial effects on SMU reduction on sleep duration.*

*
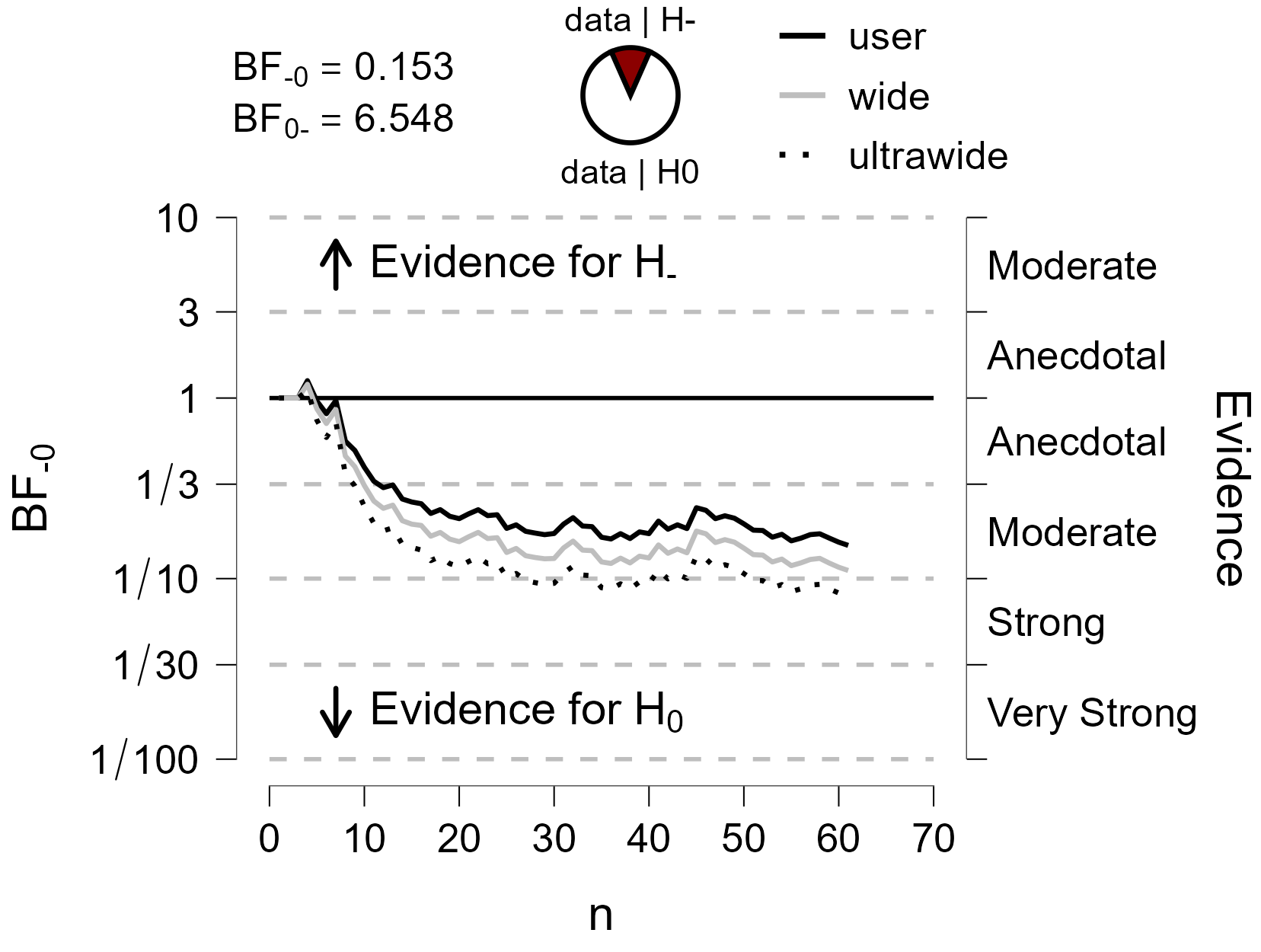
*

*Supplemental Figure 10. Cumulative evidence for the alternative vs. null-hypothesis of (no) beneficial effects on SMU reduction on actual time awake.*

*
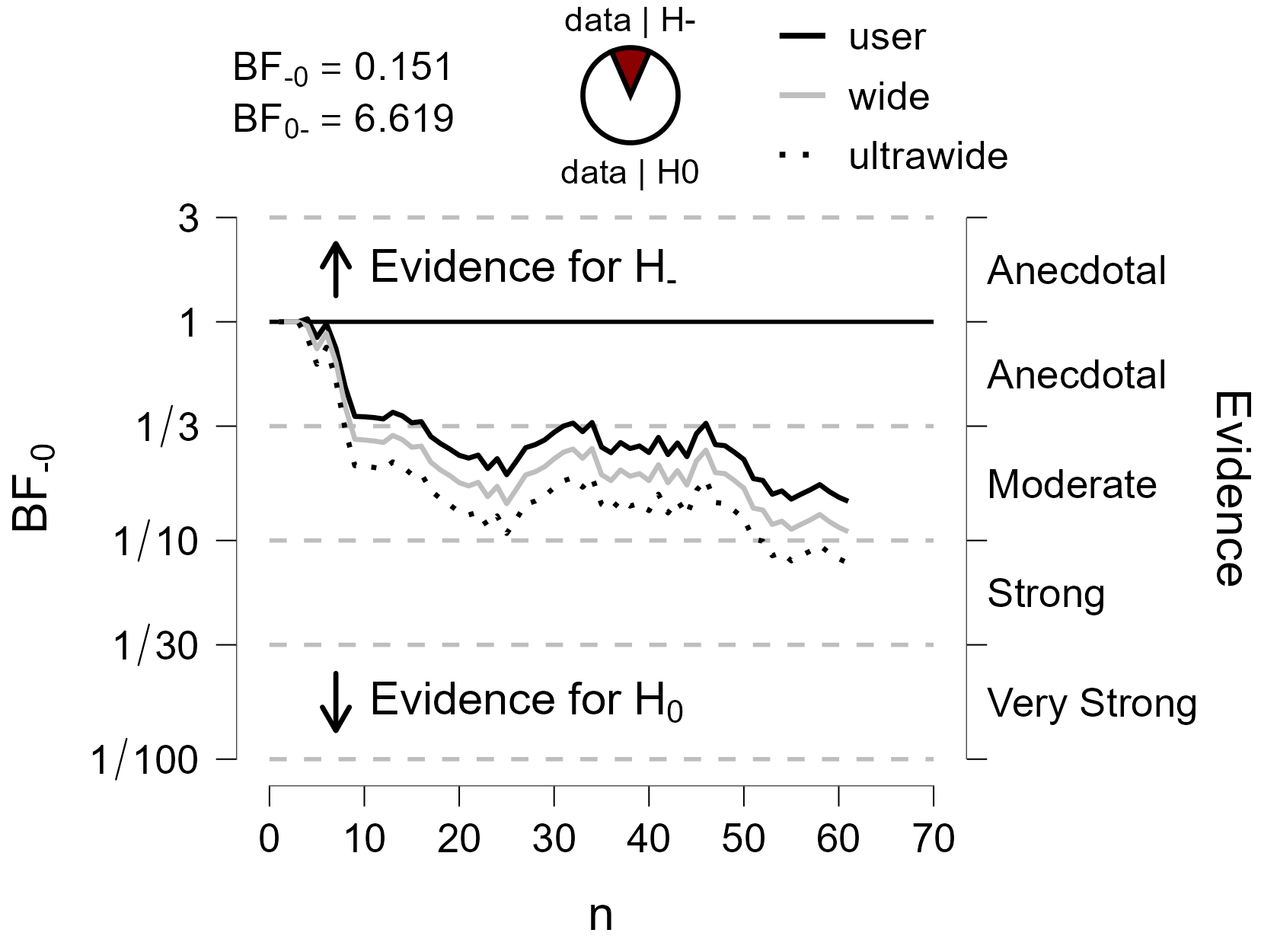
*

*Supplemental Figure 11. Cumulative evidence for the alternative vs. null-hypothesis of (no) beneficial effects on SMU reduction on sleep efficiency.*

*
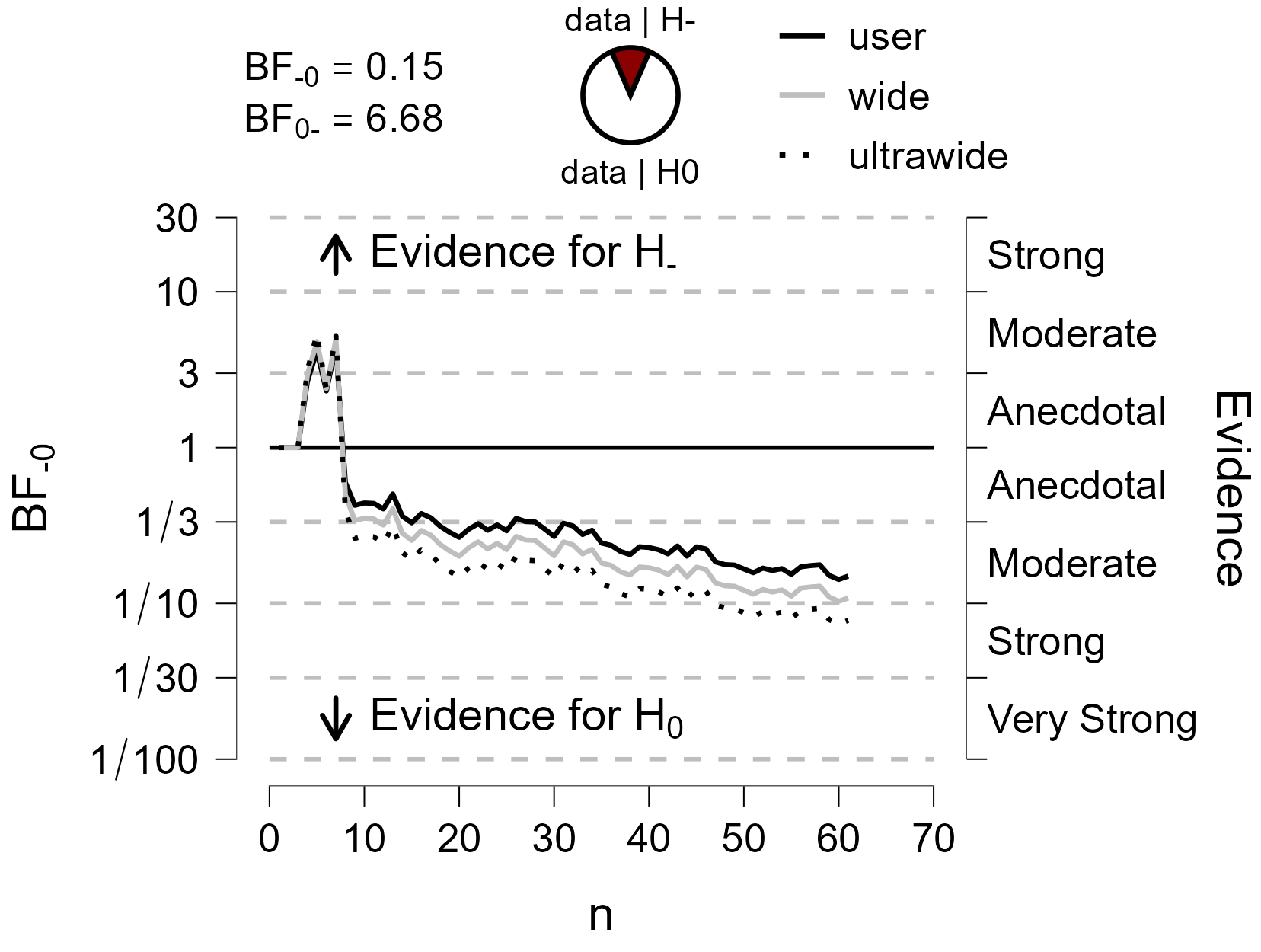
*

*Supplemental Figure 12. Cumulative evidence for the alternative vs. null-hypothesis of (no) beneficial effects on SMU reduction on fragmentation index.*
